# *AutoLEI*: An *XDS*-based pipeline with graphical user interface for automated real-time and offline batch 3D ED/microED data processing

**DOI:** 10.1107/S2052252525010784

**Published:** 2026-01-01

**Authors:** Lei Wang, Yinlin Chen, Emma Scaletti Hutchinson, Pål Stenmark, Gerhard Hofer, Hongyi Xu, Xiaodong Zou

**Affiliations:** a Department of Chemistry Stockholm University 106 91Stockholm Sweden; b Department of Biochemistry and Biophysics Stockholm University 106 91Stockholm Sweden; c Research School of Chemistry Australian National University Acton Australia; d Wallenberg Initiative Materials Science for Sustainability Stockholm University SE-106 91Stockholm Sweden; Peking University, People’s Republic of China

**Keywords:** 3D electron diffraction, 3D ED, microcrystal electron diffraction, microED, electron crystallography, real-time data processing, offline batch data processing, data analysis, beam-sensitive materials

## Abstract

Three-dimensional electron diffraction (3D ED), also known as microcrystal electron diffraction (microED), is an emerging method for determining structures from submicron-sized crystals. With the development of rapid and convenient data collection protocols, acquiring dozens of datasets in a single 3D ED/microED session has become routine. A fast and automated workflow for processing, scaling and merging a large number of 3D ED/microED datasets can significantly accelerate the structure determination process. Herein, we present an *XDS*-based pipeline with a graphical user interface for automated real-time and offline batch 3D ED/microED data processing (*AutoLEI*). We demonstrate the functionality and applications of the pipeline through four examples, using both offline and real-time data processing capabilities. The samples include small organic molecules, metal–organic frameworks (MOFs) and proteins, showcasing the versatility and efficiency of *AutoLEI* in various applications.

## Abstract 2

A pipeline with a graphical user interface for real-time and offline 3D ED/microED data processing by *XDS* was developed. The pipeline aims to improve efficiency, minimize redundant data processing work and provide users with real-time feedback during data collection.

## 1. Introduction

Three-dimensional electron diffraction (3D ED), also known as microcrystal electron diffraction (microED), has been developed into a robust method for crystal structure determination over the last few decades (Kolb *et al.*, 2007 ▸; Zhang *et al.*, 2010 ▸; Shi *et al.*, 2013 ▸; Gemmi *et al.*, 2019 ▸). The method enables rapid and accurate structure determination using commercially available transmission electron microscopes (TEMs). A wide range of structures, including zeolites (Guo *et al.*, 2015 ▸; Cho *et al.*, 2023 ▸; Lu *et al.*, 2024 ▸), metal–organic frameworks (MOFs) (Huang, Willhammar & Zou, 2021 ▸; Huang, Grape *et al.*, 2021 ▸), pharmaceuticals (van Genderen *et al.*, 2016 ▸; Lightowler *et al.*, 2022 ▸) and macromolecules (Shi *et al.*, 2013 ▸; Xu *et al.*, 2019 ▸; Martynowycz *et al.*, 2022 ▸), have been successfully solved with 3D ED/microED.

Benefiting from the strong interaction between electrons and matter, 3D ED/microED data can be collected from crystals that are too small for single-crystal X-ray diffraction (Tremlett *et al.*, 2025 ▸). An ideal TEM grid typically contains hundreds of diffracting crystals or crystal fragments. With the latest 3D ED/microED protocol, it is possible to determine structures of high-symmetry stable crystals through a single dataset within a few minutes (Cichocka *et al.*, 2018 ▸; Plana-Ruiz *et al.*, 2020 ▸; Ito *et al.*, 2021 ▸; Unge *et al.*, 2024 ▸). However, owing to the inherent limitations of the goniometer, data merging for crystals of low symmetry is usually required to achieve high data completeness. For highly beam sensitive material, a small-wedge data collection and merging strategy can also be employed to increase the signal-to-noise ratio and data completeness (Clabbers *et al.*, 2021 ▸). Furthermore, selectively merging 3D ED/microED datasets can improve data quality by increasing redundancy (Xu *et al.*, 2018 ▸; Samperisi *et al.*, 2021 ▸; Bengtsson *et al.*, 2022 ▸). More recently, high-throughput data collection has been achieved using software such as *Instamatic* (Cichocka *et al.*, 2018 ▸), *Automated EM Data Acquisition with SerialEM* (de la Cruz *et al.*, 2019 ▸), *Leginon* (Cheng *et al.*, 2021 ▸), *EPUD* (Thermo Fisher Scientific, TFS) and *Latitude D* (GATAN) or a dedicated electron diffractometer, such as Synergy-ED (Rigaku) and ELDICO ED-1 (ELDICO Scientific). It is feasible to perform qualitative phase analysis by collecting and processing well populated datasets (Luo *et al.*, 2023 ▸; Unge *et al.*, 2024 ▸; Lightowler *et al.*, 2024 ▸). In all the above cases, an efficient, automated and even real-time data processing solution is highly desirable to enhance the efficiency of structure determination and phase analysis using 3D ED/microED.

A large selection of well established software, such as X-ray detector software (*XDS*) (Kabsch, 2010 ▸), *PETS2* (Palatinus *et al.*, 2019 ▸), *CrysAlisPro-ED* (Truong *et al.*, 2023 ▸), *xia2.multiplex* (Gildea *et al.*, 2022 ▸) and *DIALS* (Winter *et al.*, 2018 ▸, 2022 ▸), is available for processing 3D ED/microED data. Data processing can be performed via either command-line instructions or graphical user interfaces (GUIs) (Powell *et al.*, 2021 ▸; Brehm *et al.*, 2023 ▸), offering flexibility to users. However, format conversion, sorting, processing, analyzing and merging a large number of 3D ED/microED datasets can be time consuming and laborious. Automated batch processing and real-time processing capabilities are still underdeveloped for 3D ED/microED. In our previous work, *Edtools* (Wang *et al.*, 2019 ▸) was developed to process batch datasets in offline mode, which only works in a command line. A processing pipeline *Scipion-ED* (Bengtsson *et al.*, 2022 ▸) embedded in *Scipion* was also developed. However, the complex operation and specialized platform requirements make these programs difficult for beginners to use.

In this work, we utilized *XDS* as the data processing engine and developed a pipeline with a graphical user interface for automated real-time and offline batch 3D ED/microED data processing (*AutoLEI*). Based on the fast data processing performance of *XDS*, *AutoLEI* enables both real-time and offline batch 3D ED/microED data processing. This Python-based pipeline is straightforward to install and provides users with the options and parameters that are essential for working with electron diffraction data. Meanwhile, data processing statistics and quality indicators are also optimized for 3D ED/microED. Here, we demonstrate how to use *AutoLEI* to process data collected from different types of crystals, including an organic molecule, a metal–organic framework and two proteins, on different microscopes and detectors. An example of real-time data processing using *AutoLEI* is also presented. Furthermore, a detailed tutorial with training datasets is provided.

## 2. *AutoLEI* implementation

### 2.1. *AutoLEI* installation and supported data formats

*AutoLEI* is an open-access, Python-based 3D ED/microED data processing pipeline, available through PyPI, GitLab and Zenodo. The software operates on Linux systems or within the Windows Subsystem for Linux (WSL) environment with Python version 3.8 or greater, and requires *XDS* to be pre-installed and configured globally. *AutoLEI* also provides a one-click installation command that automatically installs all required dependencies on GitLab. Detailed installation instructions are available on corresponding GitLab wiki (see *Data availability*). In version 1.0.0, the native image format is SMV for *XDS* data processing. *AutoLEI* can convert MRC images collected by *EPUD* (TFS), and TIFF images collected on CheeTah (Amsterdam Scientific Instruments, ASI), OneView (GATAN) and Timepix hybrid pixel detectors (ASI). Other formats need to be converted in advance to SMV format using other software, for example *REDp* (Wan *et al.*, 2013 ▸) and *RosettaSciIO* (Prestat *et al.*, 2025 ▸). Real-time data processing is designed for raw MRC image data collected using *EPUD* (TFS) as well as data collected using *Instamatic* (Cichocka *et al.*, 2018 ▸). Future updates are expected for improved compatibility and increased support, while users can also add new functionality through the programming interface.

### 2.2. *AutoLEI* data processing workflow

**Figure 1:**
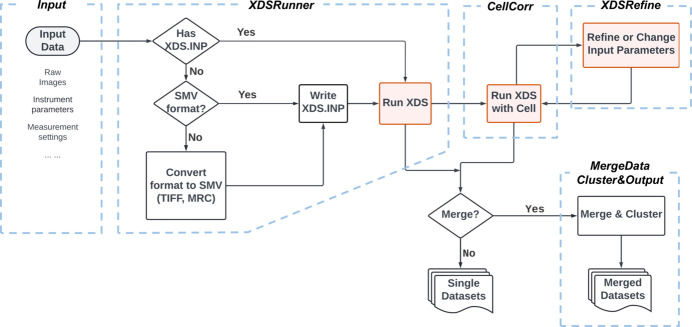
An overview of the *AutoLEI* workflow.

The primary aim of *AutoLEI* is to process and analyze a batch of 3D ED/microED datasets using *XDS* offline or in real time. *AutoLEI* builds upon the experience from our earlier software, *Edtools* (Wang *et al.*, 2019 ▸). While *Edtools* relies on command-line input and requires an existing XDS.INP file generated by *Instamatic* (Cichocka *et al.*, 2018 ▸), *AutoLEI* introduces a graphical user interface (GUI) and supports image data collected using various acquisition software. Moreover, *AutoLEI*’s workflow has been specifically optimized for rotational electron diffraction data, allowing the processing to be significantly accelerated and enabling efficient real-time analysis. Fig. 1 ▸ shows the workflow of *AutoLEI*. Data processing goes through the steps of *XDSRunner*, *CellCorr*, *XDSRefine*, *MergeData* and *Cluster&Output*. These steps are implemented as tabs in the *AutoLEI* interface, as shown in Figures S1–S8 in the supporting information. By following this workflow, final HKL files are obtained for downstream structural determination. Videos 1–3 in the supporting information showcase the offline data processing of three examples: tyrosine, MOF SU-100 and the protein *H. sapiens* MutT homolog 1 (MTH1) while Video 4 shows the real-time data processing of triclinic lysozyme.

#### 2.2.1. Input

*Input* allows users to set up the work directory, instrument parameters and measurement settings (Figure S1). The instrument parameters can be loaded from a predefined instrument model or existing input files. Alternatively, they can be given manually when datasets collected from different instruments and detectors are used. *Input* creates the Input_parameters.txt file (see an example in Section S3.2 of the supporting information), which will be used for generating input files XDS.INP for both real-time and offline 3D ED/microED data processing.

#### 2.2.2. XDSRunner

*XDSRunner* generates the corresponding XDS.INP files for each dataset (Figure S2). It then launches *XDS* to process these datasets. No prior knowledge of the sample is required. *AutoLEI* processes all datasets sequentially under the work directory and parses statistics from *XDS* outputs. After data processing, *AutoLEI* will generate a summary table named xdsrunner.xlsx. The table contains key parameters and quality indicators such as space group (SG), unit-cell parameters (Unit cell), cell volume (Vol.), indexing rate (Index%), *I*/σ(*I*)^asymptotic^ (ISa), redundancy-independent merging *R* factor (*R*_meas_), correlation between reflection intensities of random half datasets (CC_1/2_), completeness and estimated resolution (Reso.) of each dataset for inspection (Diederichs, 2010 ▸; Karplus & Diederichs, 2012 ▸). SG, unit cell and ISa are read from *XDS* outputs directly while others are calculated by *AutoLEI* based on *XDS* HKL files. The high-resolution cut-off of each dataset is estimated by checking CC_1/2_, *R*_int_ and the signal-to-noise ratio of each resolution shell.

*XDSRunner* also contains supporting functions as described below:

*Format converter*: Converts raw image files to SMV format and writes the metadata into SMV files.

*Find beam center*: Finds the beam center automatically with or without a beam stop.

*Estimate symmetry & cell-cluster*: Clusters data based on unit-cell parameters and estimates the Laue group. The Laue group is estimated based on *R*_meas_ and CC_1/2_ using four indicators. The unit-cell clustering is performed based on a distance matrix. These ensure that data are processed in the correct Laue group. Details of these functions can be found in Section S1.1 of the supporting information.

#### 2.2.3. CellCorr

*CellCorr* allows users to run *XDS* with given unit-cell parameters and space group (Figure S3). It allows re-indexing of the original HKL file using the space group and unit-cell parameters suggested by either *Estimate symmetry & cell-cluster* in *XDSRunner* or determined by other software packages such as *REDp* (Wan *et al.*, 2013 ▸) or *PETS2* (Palatinus *et al.*, 2019 ▸). This ensures that the datasets will be processed, scaled and merged in the correct Laue group and unit cell. *CellCorr* outputs a summary table named xdsrunner2.xlsx containing the same data quality indicators as xdsrunner.xlsx.

#### 2.2.4. XDSRefine

*XDSRefine* provides the opportunity to update *XDS* keywords in the input files for single, selected or all datasets if users are not satisfied with the outputs from *CellCorr* (Figure S4). The step is useful for problematic data that cannot be processed using default settings. In *AutoLEI* version 1.0.0, *XDSRefine* also provides automatic refinement of the rotation axis, and excludes problematic frames defined by outliers; see Section S1.2 for more details. The reconstructed reciprocal lattice of each dataset can also be visually inspected. *XDSRefine* updates xdsrunner2.xlsx and the table will then be used for downstream data scaling and merging.

#### 2.2.5. MergeData

*MergeData* analyzes statistics in xdsrunner2.xlsx and makes initial selections of datasets for merging (Figure S5). Users can then inspect key indicators listed in the table and apply a filter for scaling/merging. The default value will be provided as a reference. The information about the filtered data is written to the file xdspicker.xlsx. *MergeData* will then initiate *XSCALE* and output scaled data as an HKL file.

#### 2.2.6. Cluster&Output

*Cluster&Output* performs cluster analysis based on pairwise correlation coefficients of reflection intensities in the file XSCALE.Lp generated from the last step (Figure S6). A dendrogram is produced for visual inspection for further clustering (Wang *et al.*, 2019 ▸). *Cluster&Output* will then perform scaling/merging of each cluster, outputting corresponding HKL files and data processing statistics (Figure S7). Web-page-based reports will also be generated for inspection of the data reduction.

### 2.3. Real-time processing

**Figure 2:**
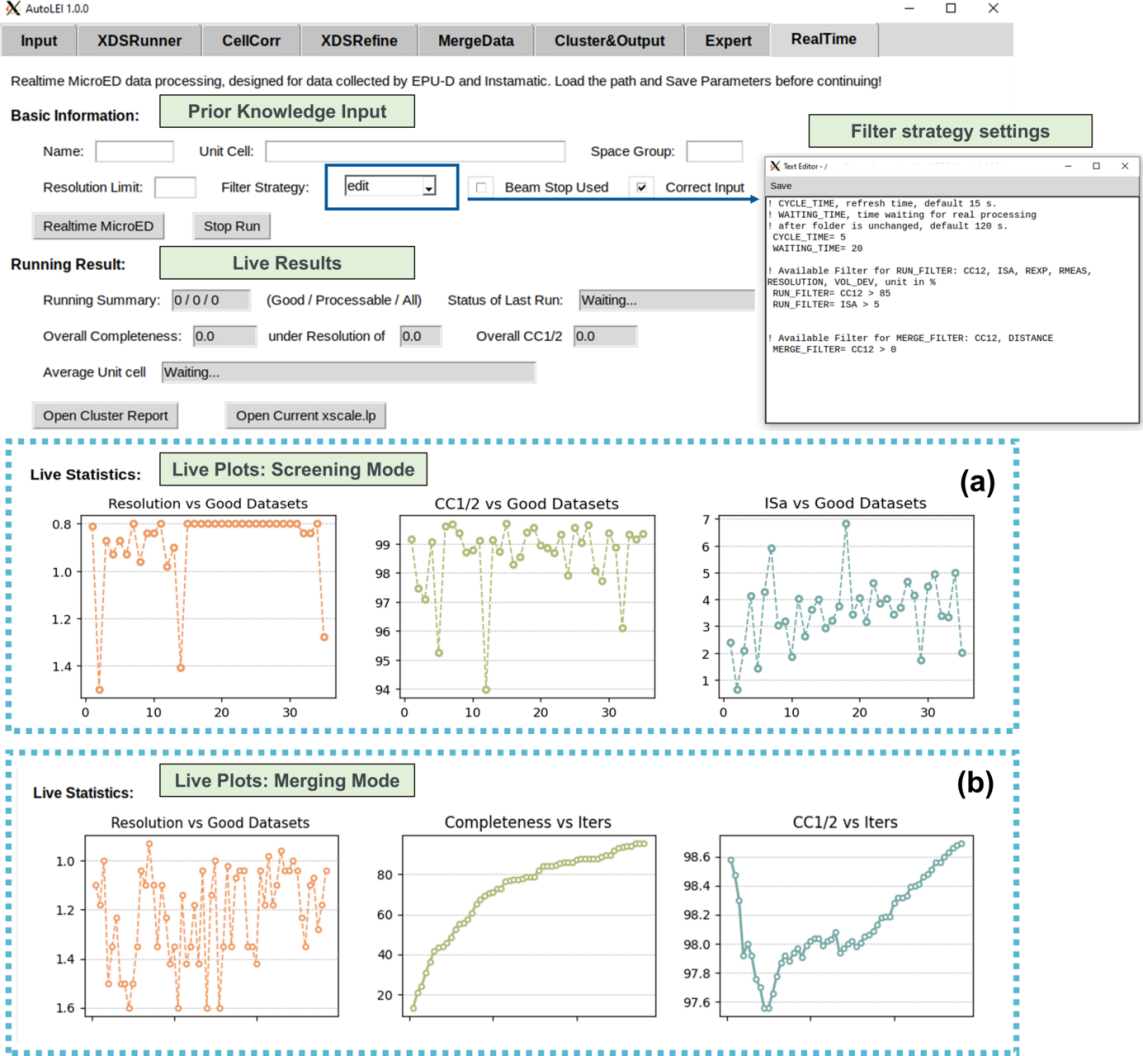
*RealTime* microED work page. (*a*) Screening mode. (*b*) Merging mode. ‘Iters’ in (*b*) denotes the number of merging iterations.

Real-time 3D ED/microED data processing is a fully automated pipeline in *AutoLEI* (Figure S8). With default or customized settings, it provides crucial feedback to users for data quality analysis, evaluates data collection parameters and generates result HKL files in real time. The setup of the real-time processing function is found on the *RealTime* tab of *AutoLEI*, as shown in Fig. 2 ▸. Real-time processing reads Input_parameters.txt from *Input* and continuously monitors the input folder into which the data are being saved. Once a single dataset collection is completed, real-time processing will try to process the data in either screening mode or merging mode:

(*a*) *Screening mode*: When the unit-cell parameters and space group are not provided, the data are processed without performing data merging.

(*b*) *Merging mode*: When the unit-cell parameters and space group are provided, the data will be processed in the specified cell and space group. If the data quality satisfies the filtering condition, it will be assigned as a valid dataset and merged with other valid datasets.

In the *RealTime* tab, estimated resolution, CC_1/2_ and ISa for individual data sets or merged data are plotted and updated live, as shown in Figs. 2 ▸(*a*) and 2(*b*), respectively. Real-time processing currently supports the data collected using *EPUD* (TFS) and *Instamatic* (Cichocka *et al.*, 2018 ▸).

## 3. Examples

**Figure 3:**
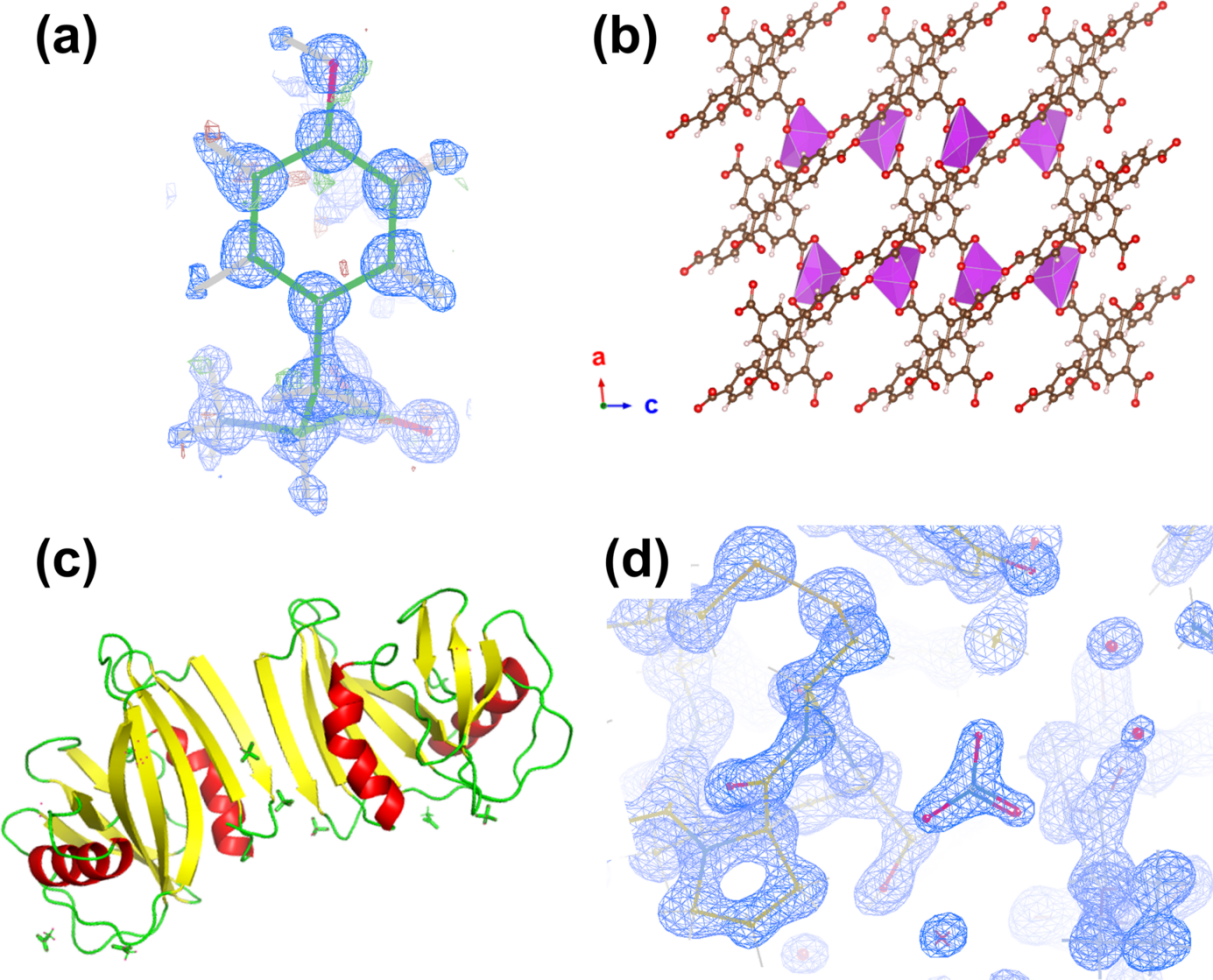
Structures used for the demonstration of *AutoLEI*. (*a*) l-tyrosine obtained using a selected high-quality dataset. The electrostatic potential map (in blue, contoured at 1 RMSD) and difference map [positive in green and negative in red, contoured at 3 RMSD drawn in *COOT* (Emsley *et al.*, 2010 ▸)] are superimposed. (*b*) The framework of the bismuth-containing MOF SU-100 viewed along the *b* axis (BiO_7_ polyhedra shown in purple). (*c*) Apo-MTH1. (*d*) Lysozyme model near a nitrate anion with no-filled 2*F*_o_ − *F*_c_ electrostatic potential map (blue, contoured at 1.5 RMSD) superimposed.

To demonstrate the functions and applications of *AutoLEI*, we chose four different samples (Fig. 3 ▸): an organic compound, tyrosine (*P*2_1_2_1_2_1_, *a* = 6.92 Å, *b* = 21.15 Å, *c* = 5.84 Å) (Boggs & Donohue, 1971 ▸), a bis­muth metal–organic framework, SU-100 (*I*2/*a*, *a* = 17.85 Å, *b* = 9.61 Å, *c* = 21.05 Å, β = 96.77°) (Grape *et al.*, 2020 ▸), a protein, MutT homolog 1 (MTH1) (*P*2_1_2_1_2_1_, *a* = 59.5 Å, *b* = 67.1 Å, *c* = 80.1 Å) (Svensson *et al.*, 2011 ▸), and triclinic lysozyme (*P*1, *a* = 27.07 Å, *b* = 31.25 Å, *c* = 33.76 Å, α = 88.0°, β = 108.0°, γ = 112.1°) (Wang *et al.*, 2007 ▸). 3D ED/microED data were collected on a variety of microscopes and detectors using different software and processed using *AutoLEI*, as summarized in Table 1 ▸. Among them, tyrosine (Video 1), SU-100 (Video 2) and MTH1 (Video 3) use the offline data processing protocol, while lysozyme (Video 4) showcases the online data processing capability. The sample preparation details are given in Section S2.

### 3.1. Rapid data quality assessment and selection of a high-quality dataset for the structure determination of tyrosine

Because of the rapid 3D ED/microED data collection, many datasets can often be collected from different crystals. It is therefore important to make a rapid data quality assessment, in particular to identify datasets with the highest and lowest quality. Here we use tyrosine crystals as an example to demonstrate the capability of *AutoLEI*.

Tyrosine is a naturally occurring amino acid and an essential protein building block. Owing to its high orthorhombic symmetry and relatively high stability under the electron beam, it is possible to determine the structure of tyrosine crystals using a single 3D ED/microED dataset collected over a large tilt range. A total of 12 tyrosine datasets were collected by *Instamatic* on a JEOL JEM2100 microscope at 200 kV using a Timepix detector. The crystal tracking function was used to ensure that most of the data were collected over a large tilt range (∼130°) (Cichocka *et al.*, 2018 ▸).

**Figure 4:**
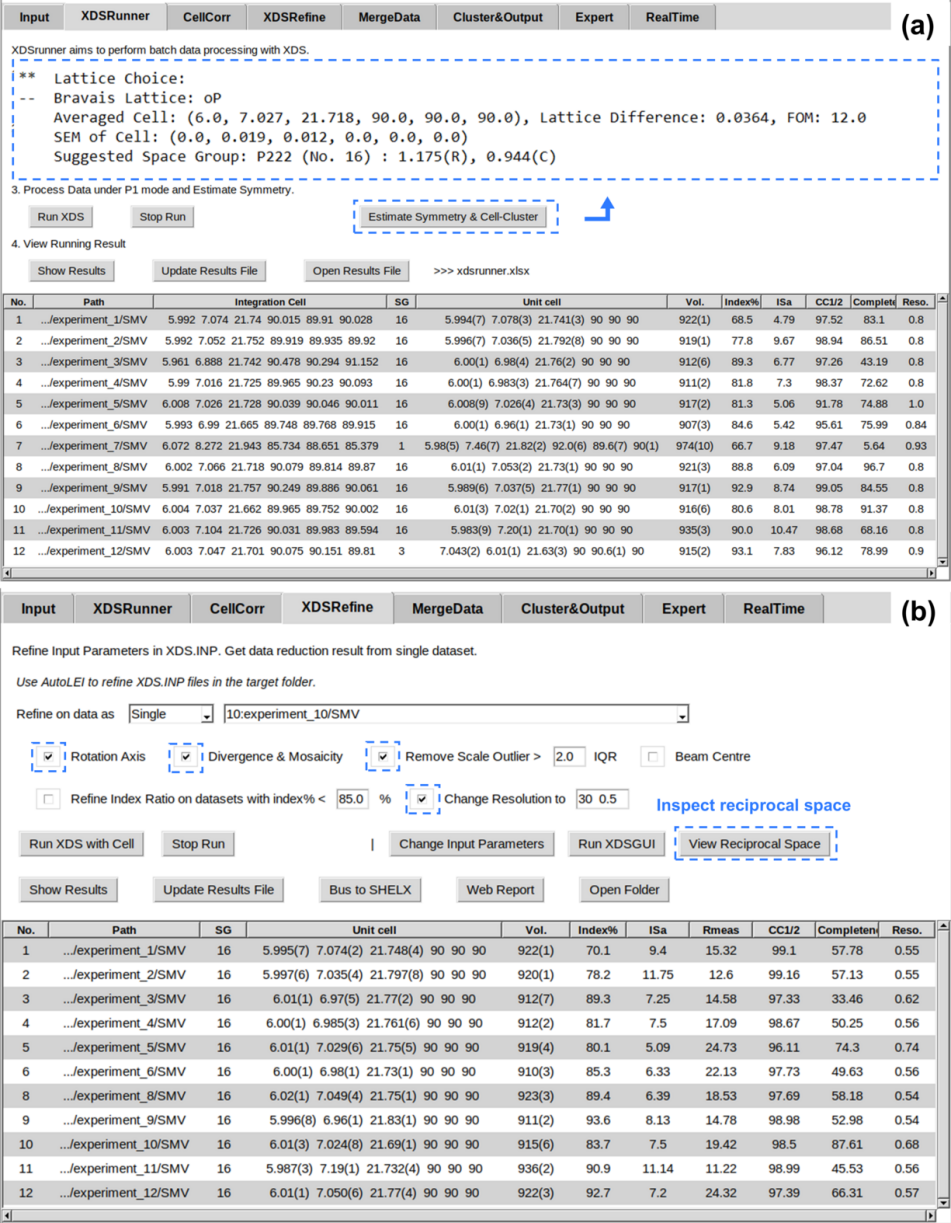
Batch data processing of 12 l-tyrosine datasets using *AutoLEI*. (*a*) Result table after running *XDSRunner*. The suggested unit-cell parameters with specific Laue group and lattice type setting were extracted from the function *Estimate Symmetry & Cell Cluster*. (*b*) Result table after running *XDSRefine*. *View Reciprocal Space* was designed to inspect the reciprocal space of a single dataset.

In the *XDSRunner* tab, *XDS* first ran without a specified space group and automatically determined the Laue group and Bravais lattice. A default resolution range from 20 to 0.8 Å was set for the initial data processing. Unit-cell parameters, space group, ISa, CC_1/2_, completeness and estimated resolution were extracted from *XDS* outputs, as shown in Fig. 4 ▸(*a*). Most of the datasets exhibit the estimated resolution of 0.8 Å, which equals the pre-defined high-resolution limit. This suggests that the resolution range should be extended. Notably, *XDS* assigned the space group *P*222 (No. 16) to 10 out of the 12 datasets while giving wrong space groups for two datasets (No. 7 and 12). By applying the built-in *Estimate symmetry & cell clustering* function – optimized for electron diffraction data – it was confirmed that these two datasets share the same Laue group and have similar unit-cell parameters to the other datasets. This allows new *XDS* runs to be performed in the *CellCorr* tab with the specified space group (*P*222) and unit cell (*a* = 6.000 Å, *b* = 7.027 Å, *c* = 21.718 Å, α = β = γ = 90°) [Fig. 4 ▸(*a*)]. Next, we applied *XDSrefine* to refine the rotation axis, divergence and mosaicity, remove scale outliers, and change the resolution range to 30–0.5 Å, as indicated in Fig. 4 ▸(*b*). After the refinement, statistic values such as CC_1/2_ and estimated resolution are improved for most of the datasets. For example, the resolution of experiment 5 is improved from 1.0 to 0.74 Å, where the CC_1/2_ increases from 91.78% to 96.11%.

The choice of optimal datasets can vary depending on which key indicators are prioritized. From our experience, we recommend datasets with ISa > 5, CC_1/2_ > 95% and completeness > 80% as being promising for single-data-set-based structure determination. Here we selected experiment 10 because it has the highest completeness (87.61%) and also a high ISa (7.51), CC_1/2_ (98.18%) and resolution (0.68 Å). *AutoLEI* also enables the systematic absences in reciprocal space to be visually inspected. By rapidly checking the reciprocal lattices of all datasets using *AutoLEI*, the presence of systematic absences could be identified on *h*00, 00*l* in experiment 10 and 0*k*0 in experiment 12 (Figure S9). The space group was deduced as *P*2_1_2_1_2_1_. The time used for data processing and analysis was approximately 5 minutes in total. The integrated data were then used for structure solution and kinematic refinement. The refinement converged to a final *R*_1_ of 11.90% for 1253 reflections with *I* > 2σ(*I*) and 162 parameters (Table S1). All hydrogen atoms, including the proton near the N atom, could be found as shown in Fig. 3 ▸(*a*). The structure could also be solved and refined using other datasets (Table S1) except for two datasets (experiment 3 and experiment 7) that had too low completeness (43.19% and 5.64%, respectively) [Fig. 4 ▸(*a*)] due to beam blockage by the grid.

In conclusion, *AutoLEI* can directly generate data information tables so that users can make their own assessment of the data quality. This can help users to identify the best datasets for structure determination or to decide whether data merging is needed in cases where the completeness is low. It can also help to eliminate low-quality datasets.<!?tpb=-6pt>

### 3.2. Interactive clustering and merging of multiple datasets from a monoclinic bis­muth metal–organic framework, SU-100

**Figure 5:**
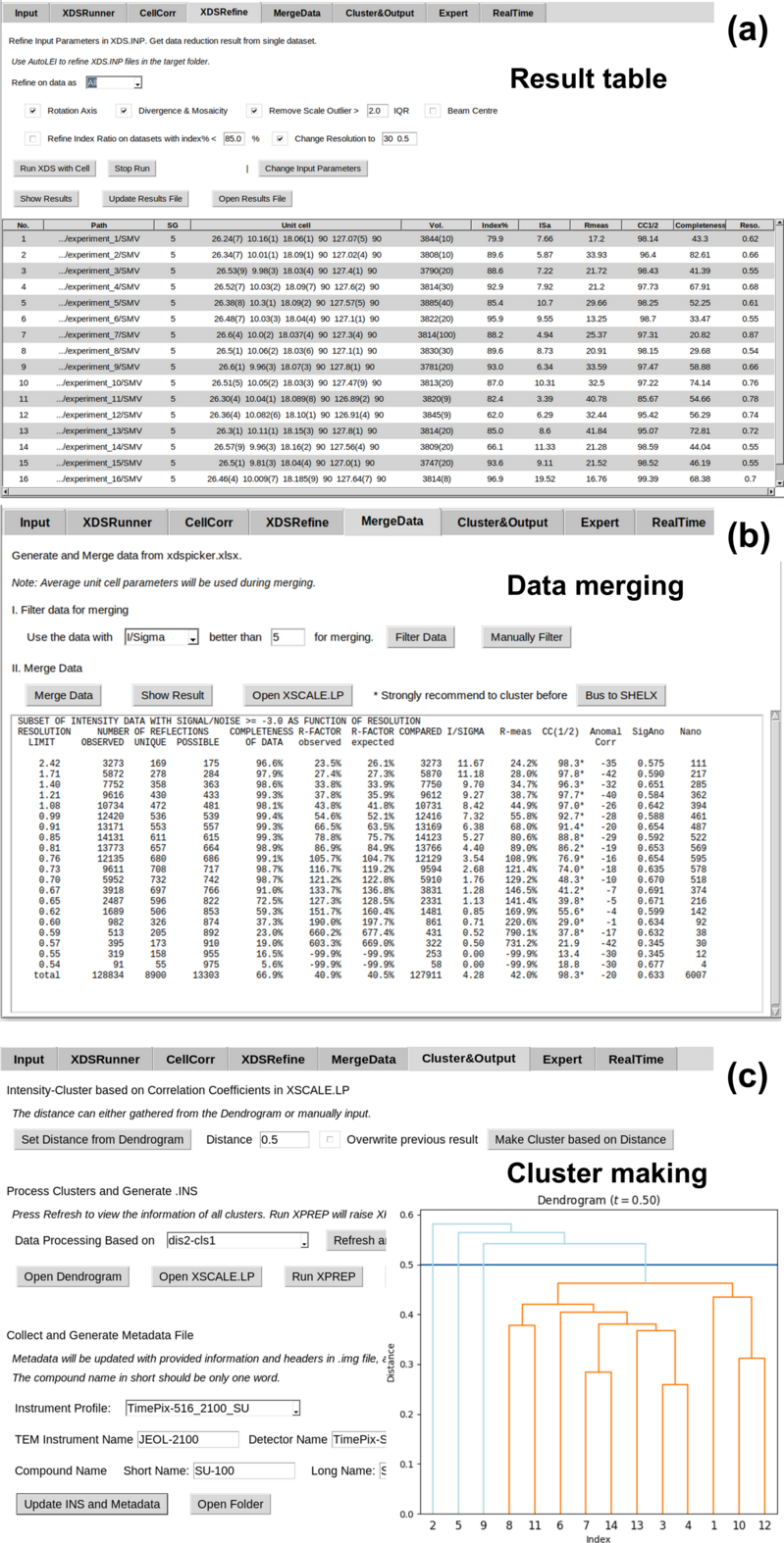
Data processing, analysis and merging of datasets of SU-100 by *AutoLEI*. (*a*) *XDSRefine* work page showing the result table of 16 datasets. (*b*) *MergeData* work page. Fourteen out of 16 datasets with ISa > 5 were used for initial merging after several clicks. (*c*) *Cluster&Output* work page. Clusters were created based on pairwise reflection intensity correlations and a final HKL file for structure determination was prepared that contained merged data from the largest cluster of 11 datasets (Wang *et al.*, 2019 ▸).

For crystals with low symmetry, merging datasets collected from crystals from different orientations is often needed to achieve high completeness. In addition, data merging can minimize the effects of dynamical scattering and improve the data quality (Xu *et al.*, 2018 ▸). Here we chose a monoclinic bis­muth metal–organic framework (*I*2/*a*, *a* = 17.85 Å, *b* = 9.61 Å, *c* = 21.05 Å, β = 96.77°) (Grape *et al.*, 2020 ▸). In this study, a total of 16 datasets were collected using *Instamatic*, and all data were processable after *XDSRefine*, as shown in Fig. 5 ▸(*a*). The data resolution ranges from 0.54 (experiment 8) to 0.78 Å (experiment 11), and the completeness ranges from 20.82% (experiment 7) to 82.61% (experiment 2). Fourteen out of the 16 datasets have an ISa higher than 5 and were then merged during the *XDSRefine* step after specifying the space group and unit-cell parameters [Fig. 5 ▸(*b*)]. We further conducted cluster analysis based on the pairwise correlation coefficients of reflection intensities (Wang *et al.*, 2019 ▸). As shown in Fig. 5 ▸(*c*), a threshold of 0.5 was selected that gives a significant distance between the four clusters. The final merged HKL file from 11 datasets was obtained in 7 minutes. The resolution cut-off of the merged data estimated by *AutoLEI* was 0.62 Å. All non-H atom positions could be directly located and refined anisotropically using the merged data. The refinement converged to an *R*_1_ of 19.46% for 4231 unique reflections with *I* > 2σ(*I*) and 200 parameters, as shown in Table S2.

It is important to mention that data merging can significantly improve the data quality (Xu *et al.*, 2018 ▸; Samperisi *et al.*, 2021 ▸). We compared the structure refinement using the merged dataset and a single dataset from experiment 2, which has the highest completeness of all single datasets, as shown in Figure S10. While the structure could be refined anisotropically using the merged data, anisotropic refinement using a single dataset of experiment 2 gave non-positive-definite atomic displacement parameters for 19 atoms.

### 3.3. Processing small-wedge 3D ED/microED data from the protein MutT homolog 1 (MTH1)

For structure determination of very beam sensitive crystals, such as protein crystals (Shi *et al.*, 2013 ▸), a small-wedge data collection strategy (Clabbers *et al.*, 2021 ▸) can be used, where a large number of 3D ED/microED datasets are collected from different crystals, each with a small tilt range (10–20°) and a low completeness. *AutoLEI* is ideal for conducting repetitive data processing, data scaling and merging of such batch data.

**Figure 6:**
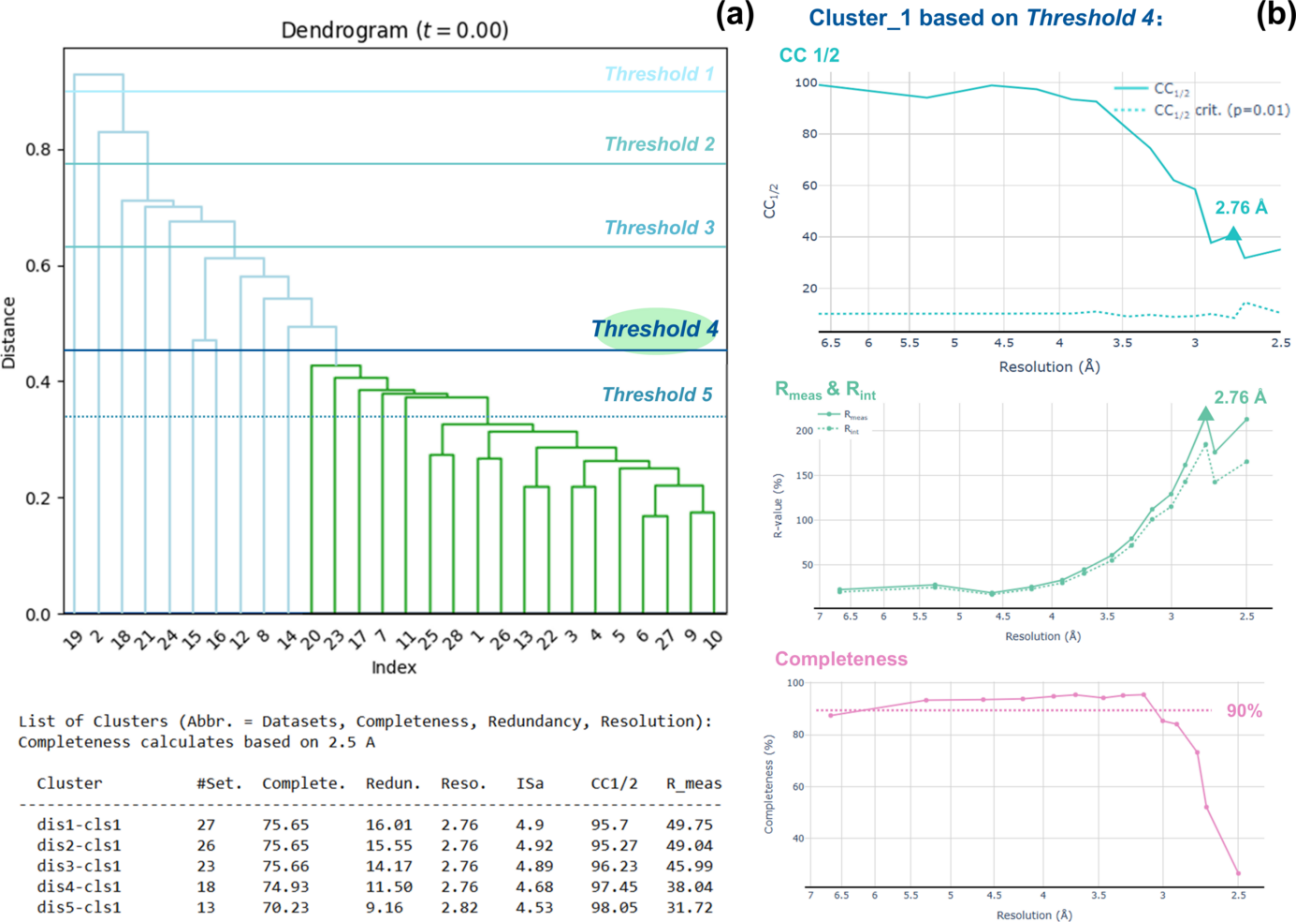
Data processing, analysis and merging of 38 MTH1 datasets by *AutoLEI*. (*a*) Clustering analysis after data merging in the *AutoLEI* interface. A total of 5 cluster selections were made based on different distances. A summary of the key statistics of these 5 clusters was tabulated in the command window. (*b*) Web-page-based report plots of Cluster_1 based on threshold 4 (18 datasets in total). Plots were captured from the report. From top to bottom: CC_1/2_ versus resolution, *R*_meas_ and *R*_int_ versus resolution, and completeness versus resolution.

MTH1 plays an important role in sanitizing oxidized dNTPs from the free nucleotide pool, preventing their incorporation into DNA, which reduces genotoxicity (Yoshimura *et al.*, 2003 ▸; Gad *et al.*, 2014 ▸; Jemth *et al.*, 2018 ▸). Here, we used apo-MTH1 as an example to demonstrate how small-wedge 3D ED/microED data can be effortlessly processed and merged with *AutoLEI*. A total of 38 datasets were collected using *EPUD* (TFS) and the tilt range was limited to 15 or 20° in order to collect data with a high signal-to-noise ratio before the crystals were damaged by the electron beam. Only 28 out of 38 datasets were processable after *XDSRefine*. Fig. 6 ▸(*a*) shows the dendrogram of the clustering analysis after the *MergeData* step in *AutoLEI*. A stepwise clustering analysis using different distance thresholds can be performed in *AutoLEI* to find the optimal one. As shown in Fig. 6 ▸(*a*), five thresholds were tested, resulting in five groups containing 27, 26, 23, 18 and 13 datasets, respectively. Each group is called dis*X*-cls*Y*, where *X* is the threshold and *Y* is the main cluster under the threshold. The first four groups showed comparable completeness (74.93–75.66%), while the last group had a slightly lower completeness (70.23%). We chose dis4-cls1 for data merging because it has the highest CC_1/2_ and lowest *R*_meas_ among the first four groups. A web-page-based report was then generated and used for data reduction of dis4-cls1. Fig. 6 ▸(*b*) indicates the relationships between CC_1/2_, *R*_meas_ and completeness against resolution drawn from the report of dis4-cls1. The estimated resolution was 2.76 Å and it took less than 12 minutes to obtain the final HKL file using *AutoLEI*. The structure of MTH1 could be solved by *Phaser* (McCoy *et al.*, 2007 ▸) and refined using *Phenix.refine* (Afonine *et al.*, 2012 ▸; Liebschner *et al.*, 2019 ▸). The final *R*_work_ and *R*_free_ converged to 21.07% and 26.82%, respectively, as shown in Table S3.

### 3.4. Real-time batch data processing for triclinic lysozyme

Real-time data processing for single-crystal X-ray diffraction is implemented at most synchrotron X-ray facilities. This capability is also desirable for 3D ED/microED, as it provides live processing statistics on the current batch data collection. This allows users to inspect the data quality in order to decide how to optimize the data collection strategy and to stop data collection when enough data are collected. By combining *AutoLEI* with automated data collection protocols, rapid structure determination and phase analysis become possible. Here, we used triclinic lysozyme crystals to demonstrate the real-time 3D ED/microED data processing capability of *AutoLEI*.

Triclinic lysozyme crystals diffract to a very high resolution (Wang *et al.*, 2007 ▸). However, triclinic lysozyme is challenging for 3D ED/microED because of its low symmetry and preferred crystal orientation. Here, a small-wedge data collection strategy was employed to increase the data resolution. The tilt range of individual crystals was restricted to 15° to keep beam damage minimal. To achieve real-time data merging (merge mode), the unit-cell parameters and space group need to be given as prior knowledge. These two input parameters can easily be obtained by first collecting a high-tilt 3D ED/microED dataset. Data with both CC_1/2_ higher than 90% and ISa higher than 5 were considered to be good and subsequently used for real-time merging iterations. These parameters could also be customized by editing the strategy file settings. Furthermore, the resolution of each good dataset as well as the statistics of merged data completeness and CC_1/2_ were updated live. Users can then use this information to check whether the current data collection strategy or data filtering strategy should be modified.

**Figure 7:**
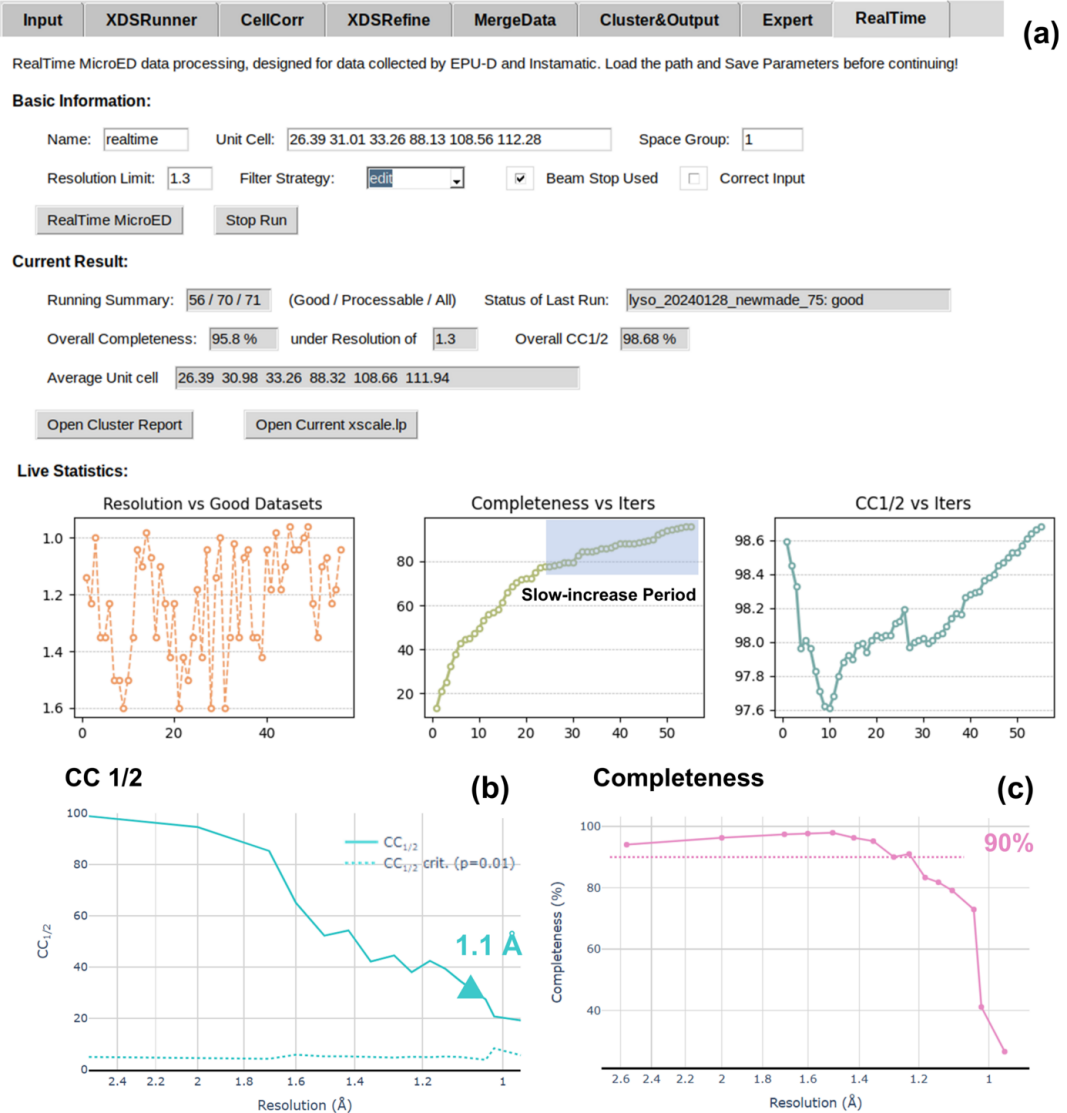
Real-time 3D ED/microED data processing of triclinic lysozyme datasets using *AutoLEI*. (*a*) The live processing results are displayed under *Live Statistics*. (*b*) CC_1/2_ and (*c*) completeness against resolution plots captured from the web-page-based report.

A total of 71 datasets were collected on lysozyme crystals in 6 h. The completeness of the data reached 95.8%, while the CC_1/2_ was 98.68% at the resolution cut-off of 1.3 Å, as shown in Fig. 7 ▸(*a*). Owing to preferred orientation, the completeness only reached 50% and 78% when merging the first 10 and 21 datasets, respectively. In order to reach a higher completeness, we had to change the data collection strategy and look for crystals with different morphologies on the TEM grids. The data collection was terminated after the merged data reached a completeness higher than 95% at the pre-set resolution cut-off of 1.3 Å and a CC_1/2_ above 98%. The web-page-based report was then generated to estimate the real high-resolution cut-off, which was set at 1.1 Å at the CC_1/2_ of 30%, as shown in Figs. 7 ▸(*b*) and 7(*c*). The final HKL file merged from 56 datasets was used immediately for structure solution using *Phaser* (McCoy *et al.*, 2007 ▸) and refinement using *Phenix* (Afonine *et al.*, 2012 ▸; Liebschner *et al.*, 2019 ▸). The final refinement converged with *R*_work_ of 20.42% and *R*_free_ of 23.45%. High *R* values are common for electron diffraction data due to dynamical effects. The no-filled 2*F*_o_ − *F*_c_ electrostatic potential map for the lysozyme structure is presented in Fig. 3 ▸(*d*).

## 4. Conclusion and outlook

We developed an *XDS*-based pipeline with a GUI, *AutoLEI*, for offline and real-time batch 3D ED/microED data processing. The visualization interface enables data import, processing, selection and merging with only a few clicks. We demonstrated the applications of *AutoLEI* on four different samples with various processing strategies to show its simplicity and ease of use. For stable or high-symmetry crystals, *AutoLEI* enables rapid identification of promising single datasets for structure determination. For low symmetry or electron-sensitive materials, *AutoLEI* can assist in selecting and merging datasets, thereby generating a final HKL file with high completeness. In addition, real-time data processing provides direct feedback, allowing users to adjust data collection strategies ‘on the fly’.

Further developments are planned for *AutoLEI*, including incorporation of additional data processing engines, enabling comparative analyses and data integration. These developments will further simplify 3D ED/microED data processing and lower the entry barriers for researchers new to the field of 3D ED/microED.

## 5. Related literature

The following references are cited in the supporting information: Burnley *et al.* (2017 ▸), Dolomanov *et al.* (2009 ▸), Giordano *et al.* (2012 ▸), Grant & Pickup (1995 ▸), Grosse-Kunstleve (1999 ▸), Grosse-Kunstleve *et al.* (2004 ▸), Jain & Dubes (1988 ▸), Knudsen *et al.* (2013 ▸), Kolb *et al.* (2012 ▸), Niggli (1928 ▸), Palatinus (2013 ▸), Schönemann (1966 ▸), Sheldrick (2008 ▸), Storn & Price (1997 ▸), Van Der Walt *et al.* (2014 ▸).

## Supplementary Material

Crystal structure: contains datablock(s) SU-100, TyrosineExp10. DOI: 10.1107/S2052252525010784/jf5003sup1.cif

Supporting information. DOI: 10.1107/S2052252525010784/jf5003sup2.pdf

AutoLEI tutorial video 1. DOI: 10.1107/S2052252525010784/jf5003sup3.mp4

AutoLEI tutorial video 2. DOI: 10.1107/S2052252525010784/jf5003sup4.mp4

AutoLEI tutorial video 3. DOI: 10.1107/S2052252525010784/jf5003sup5.mp4

AutoLEI tutorial video 4. DOI: 10.1107/S2052252525010784/jf5003sup6.mp4

Other structural data. DOI: 10.1107/S2052252525010784/jf5003sup7.zip

Raw data used for examples: https://doi.org/10.5281/zenodo.14536385

AutoLEI software: https://doi.org/10.5281/zenodo.15155987

CCDC references: 2512820, 2517260

## Figures and Tables

**Table 1:** Summary of data used for demonstrating *AutoLEI* processing JEOL: Japan Electron Optics Laboratory Co., Ltd. ASI: Amsterdam Scientific Instruments. TFS: Thermo Fisher Scientific.

Sample	Microscope	Detector	Data collection software	Total datasets	Data collection time	Data processing time†
Tyrosine (*P*2_1_2_1_2_1_)	JEM-2100 LaB_6_ (JEOL)	Timepix hybrid pixel detector (ASI)	*Instamatic*	12	∼1 h 20 min	∼5 min
SU-100 (*I*2/*a*)	JEM-2100 LaB_6_ (JEOL)	Timepix hybrid pixel detector (ASI)	*Instamatic*	16	∼2 h 15 min	∼7 min
MTH1 (*P*2_1_2_1_2_1_)	Krios G3i (TFS)	Ceta-D (TFS)	*EPUD* (TFS)	38	∼2 h 14 min	∼12 min
Lysozyme (*P*1)	Krios G2 (TFS)	Ceta-D (TFS)	*EPUD* (TFS)	72	∼6 h	real time (< 1 min)

† Timing is from the start of data processing to obtaining usable HKL files (Videos 1–3).

## Data Availability

The source code is open-source and available on GitLab (https://gitlab.com/tristonewang/autolei), Zenodo (10.5281/zenodo.15155987) and PyPI (https://pypi.org/project/autolei/). The data used as examples can be downloaded from Zenodo (https://zenodo.org/records/14536385).
