# Supplementary material for: AutoLEI: An XDS-based pipeline with graphical user interface for automated real-time and offline batch 3D ED/microED data processing
Source: IUCrJ. 2026 Jan 1;13(Pt 1):105–15. doi: 10.1107/S2052252525010784 (PMC12809502; doi:10.1107/S2052252525010784)
Supplement: Supplementary file 2 [file m-13-00105-sup2.pdf]

IUCrJ

Volume 13 (2026)

Supporting information for article:

AutoLEI: An *XDS*-based pipeline with graphical user interface for automated real-time and offline batch 3D ED/microED data processing

Lei Wang, Yinlin Chen, Emma Scaletti Hutchinson, Pål Stenmark, Gerhard Hofer, Hongyi Xu and Xiaodong Zou

S1. Supporting function implementation in AutoLEI

S1.1 *XDSRunner*

Format Transfer: The format converter leverages functions in Python libraries: mrcfile (Burnley *et al.*, 2017), FabIO (Knudsen *et al.*, 2013), and h5py (https://www.h5py.org/). SMV format does not accept negative pixel values. Consequently, during the conversion from other formats to SMV IMG, a pedestal value is systematically added to all pixels to ensure non-negative intensities. This pedestal is estimated by averaging the lowest (negative) intensity values from ten randomly selected frames within the dataset.

Find Beam Centre/Beam Stop: Depending on whether a beam stop was used, the button toggles between "Find Beam Centre" (when no beam stop was used) and "Find Beam Stop" (when a beam stop was used). This function will estimate the direct beam for each dataset with or without using beam stop during data collection and then change the input parameter of XDS input files. The beam center can also be manually determined from the intersection of lines formed by multiple Friedel pairs and specified in the Input tab before data processing begins.

Find Beam Centre: This function identifies the most intense region in the image and designates its center as the position of the direct beam.

Find Beam Stop: This function estimates the beam center from the diffuse background of the direct beam, and identifies the shadow of the beam stop. It designates the beam stop region as an untrusted area in XDS processing. Functions in Skimage (Van Der Walt *et al.*, 2014) are employed for this task.

Estimate resolution: The high-resolution cutoff of a single/merged dataset is estimated jointly by $CC_{1/2}$, R_{int} and I/Sigma. Practically, the $CC_{1/2}$ should be statistically significant, and the R_{int} should be less than 180%. When the R_{int} is within the range of 100–180%, the I/Sigma of the corresponding shell should be above 0.5.

Estimate Symmetry: To estimate the real Laue group, AutoLEI first processes the CORRECT.LP output from XDS to extract possible Bravais lattices. It then utilizes its internal HKL analyzer to calculate R_{meas} and $CC_{1/2}$ values for potential Laue groups. To facilitate the determination of the Laue group four complementary metrics are reported for each possible symmetry:

- Figure of merit (FOM) obtained from XDS
- Lattice deviation obtained from AutoLEI
- $R\_\text{ratio} = R_{\text{meas}}(\text{Laue})/R_{\text{meas}}(\bar{1})$
- $C\_\text{ratio} = [1 - CC_{1/2}(\text{Laue})]/[1 - CC_{1/2}(\bar{1})]$

To eliminate the bias from data quality, both ratios are in the quotient format. R_{meas} and $CC_{1/2}$ of the Laue group −1, which only considers the Friedel pairs, are used for reference. The (1 – $CC_{1/2}$) present

here indicates the distance between two randomly halved datasets after merging symmetry-related reflections based on that Laue group (Jain & Dubes, 1988). Both the *R*_ratio and *C*_ratio should be close to 1 for the correct Laue group or its subgroup is selected, and significantly increase when a wrong Laue group is used. Thus, the most possible Laue group shall be the highest Laue group with reasonable *R* ratio and *C* ratio.

Cell-Cluster: The unit cell setting chosen initially by XDS is arbitrary. For clustering based on the unit cell, eliminating this ambiguity is very important. Niggli reduction is often applied for this purpose, transforming the cell parameters into a unique, standardized form defined by rigorous mathematical conditions (Niggli, 1928). However, due to experimental errors or hidden higher symmetries, the same crystal can sometimes be reduced to significantly different standard cells, especially when the lattice parameters are close to those of a higher-symmetry crystal system (Grosse-Kunstleve, 1999). In AutoLEI, we addressed these challenges by performing unit-cell clustering on cells with the highest possible Laue symmetry.

After *Estimate Symmetry*, the possible Laue group with its unit cell setting is obtained. Before calculating the distance between unit cells, Niggli reduction is first performed based on the methodology reported by Grosse-Kunstleve *et al.* (2004). To minimize the influence of measurement errors, the reduced unit cell is then transformed into a matrix $\mathbf{M} = (\boldsymbol{a}^*, \boldsymbol{b}^*, \boldsymbol{c}^*)^{\mathrm{T}}$, describing the reciprocal lattice. Then orthogonal Procrustes analysis (Schönemann, 1966) is applied to calculate the distance between two matrixes M_1 and M_2. To prevent over scaling of the reciprocal cell matrix during orthogonal Procrustes analysis, the distance between unit cells is adjusted by the ratio of their volumes in an exponential format (Grant & Pickup, 1995). The final distance D is defined as:

$$D = d(\text{Procrustes}, M_1, M_2) \times \exp\left(\frac{V_1}{V_2}\right), \text{where } V_i = \frac{1}{\det M_i}, V_1 > V_2$$

The resulting distance matrix is then used for cluster analysis. For reporting purpose, all possible Laue groups and Bravais lattices are summarized within each cluster, and their metrics are averaged.

S1.2 *XDSRefine*

Rotation Axis: The algorithm is adopted with enhanced efficiency and accuracy from *edtools* (https://github.com/instamatic-dev/edtools), using its original code reported by Kolb *et al.* (2012).

Divergence and Mosaicity: Those parameters are extracted from INTEGRATE.LP. The average value is calculated by excluding the outliers. The resulting values are added to XDS.INP for the next round of data processing.

Remove Scale Outlier: The scale of each frame is extracted from INTEGRATE.LP. Outlier (values beyond a user-defined multiple of the interquartile range, IQR) of the scale may indicate that the crystal has moved out of the selected area aperture or electron beam. The corresponding frames are therefore excluded from further data processing.

Beam Centre: An algorithm is developed to load reflection positions/intensities from SPOT.XDS and then estimate the beam center. We developed an algorithm to maximize the number of Fridel pairs, taking into account the intensity difference and positional variance between assigned Fridel pairs, refined by the differential evolution method (Storn & Price, 1997).

Index Ratio: This function runs IDXREF with different parameters and uses the best combination of input parameters to maximize the indexing ratio.

View Reciprocal Space: This function reads SPOT.XDS from XDS and reconstructs reciprocal space without correction of the Ewald sphere.

S1.3 *MergeData*

Merge data: In the AutoLEI workflow, all reflections are retained for individual datasets during data reduction. A resolution cutoff, which is set to the resolution limit calculated by AutoLEI, is applied at the merging stage to ensure that reflections with low-reliability are excluded from the final HKL file.

S1.4 *Cluster&Output*

Intensity-Cluster: The distance is calculated from the correlation coefficient between datasets in XSCALE.LP obtained from XSCALE. The algorithm was described in a previous publication (Giordano *et al.*, 2012).

$$d = \sqrt{1 - CC^2}$$

S2. Experiments

S2.1 Sample preparation for tyrosine and Bi-MOF SU-100

Commercial L-tyrosine powder (Sigma-Aldrich) was ground between two glass plates. The crushed powder was then loaded onto an R2/2 Cu300 Quantifoil TEM grid.

SU-100 was synthesized as previously described (Grape *et al.*, 2020). The as-synthesized SU-100 powder was first crushed using a mortar and then dispersed in water. The R2/2 Cu300 Quantifoil TEM grid was rapidly dipped into a 1% Tween-20 solution to make the TEM grid hydrophilic. Filter paper was used to remove the extra Tween-20 solution by back-blotting. The SU-100 suspension was then transferred onto the treated TEM grid.

S2.2 Sample preparation for MTH1 and lysozyme proteins

The expression and purification of protein MTH1 were performed as described previously (Svensson *et al.*, 2011). The precipitant solution used for MTH1 crystallization contained 30% PEG6000, 0.1 M sodium acetate buffer (pH 4) and 0.16 M lithium sulfate. After mixing 0.5 μL protein solution (14 mg/mL) and 0.5 μL precipitant solution, needle-like crystals were formed within 30 min. 1 μL of these crystals was then mixed with 15 μL of precipitant solution and ground into small fragments. 15 μL of protein solution was then added to the mixture to form the micro-sized crystals used in this study.

Lyophilized lysozyme powder (Sigma-Aldrich, 62970) from chicken egg white was dissolved in 50 mM sodium acetate buffer (pH 4.5) to make a 20 mg/mL protein solution. A precipitant solution containing 0.4 M sodium nitrate and 50 mM sodium acetate (pH 4.5) was also prepared. For crystallization, 30 µL of both protein solution and precipitant solution were added to a PCR tube. The tube was sealed with parafilm and stored first overnight at 4 °C (Wang *et al.*, 2007), and then at room temperature. After 3–4 days, several X-ray-sized crystals could be observed in the PCR tube. To obtain crystals used for MicroED data collection, these crystals were ground by iron beads on a vortex mixer to produce a seed solution. 1 µL of seed crystal slurry was then added to 20 µL of precipitant solution. Microcrystals were formed by mixing the mixture with 20 µL of protein solution.

The grids for both MTH1 and lysozyme were prepared as follows: the R1.2/1.3 Cu300 Quantifoil grid was first dipped into a 1% Tween-20 solution and back-blotted using filter paper to make the carbon supporting film hydrophilic. A 1.5 µL drop of crystal slurry was then pipetted onto the treated TEM grid. The grid was then back-blotted again to remove excess liquid and plunge frozen in liquid ethane. The grids were then clipped and transferred into the autoloader of a Titan Krios cryo-TEM (Thermo Fisher Scientific) under cryogenic conditions.

S2.3 Data collection

MicroED data of tyrosine and SU-100 were collected using a JEOL JEM-2100 transmission electron microscope equipped with a Timepix detector (Amsterdam Scientific Instrument), operated at 200 kV. A Gatan 914 cryo-transfer holder was used to collect data at cryogenic temperature (100 K). *Instamatic* (Cichocka *et al.*, 2018) was used for high-throughput data collection with crystal tracking and metadata recording. The tilt step for data collection was 0.23°/frame and the exposure time was 0.5 s/frame. The tilt range is reported in the AutoLEI report.

MicroED data collection of MTH1 protein micro-crystals was performed using a Titan Krios G3i microscope (300 kV) while lysozyme data were collected on a Titan Krios G2 microscope (300 kV). Both microscopes were equipped with an autoloader for sample loading and a Ceta-D detector for data collection. EPUD was used for data collection. In both cases, data were collected under nanoprobe mode, without the use of a selected area aperture. The beam size and flux used for data collection were 1 µm and 0.14 $e^{-}/Å^{2}/s$ for MTH1 and 1.5 µm and 0.12 $e^{-}/Å^{2}/s$ for lysozyme. The tile step used for MTH1 data collection was 1 °/frame and the exposure time was 1 s/frame. The tilt range for each dataset was 15 or 20°. For lysozyme, the tilt step and exposure time were set to 0.5 °/frame and 1 s/frame. The total tilt range for each dataset was 15 °.

S2.4 Structure determination

Structure determination of all L-tyrosine data and merged SU-100 data was performed in Olex2 (Dolomanov *et al.*, 2009) using SHELXS for structure solution and SHELXL for structure refinement (Sheldrick, 2008). For experiment_2 in the SU-100 study, the initial phases were calculated by charge flipping (Palatinus, 2013). The structures of MTH1 and lysozyme proteins were determined using Phenix (Liebschner *et al.*, 2019). Molecular replacement was carried out using Phaser (McCoy *et al.*,

2007) and the structure refinement was performed with Phenix.refine (Afonine *et al.*, 2012; Liebschner *et al.*, 2019).

S3. Example of Files through Data Processing

S3.1 Instrument Profile:


```
{
  "instrument": "JEOL-2100",
  "detector": "TimePix-SU",
  "NX": 516,
  "NY": 516,
  "QX": 0.055,
  "QY": 0.055,
  "overload": 65000,
  "rotation_axis": -128.34,
  "energy": 200,
  "wavelength": 0.02510,
  "addition information": [
    "UNTRUSTED_RECTANGLE= 255 262 0 517",
    "UNTRUSTED_RECTANGLE= 0 517 255 262"
  ]
}
```


S3.2 Input Parameter File:

```
###Uniform Experiment Settings###
1. Pixel information for your camera:
 NX= 516   NY= 516  QX= 0.055  QY= 0.055  !Number and Size (mm) of pixel

2. Overload range for your camera:
 OVERLOAD= 65000   !default value dependent on the detector used

3. Resolution range for the 1st round:
 INCLUDE_RESOLUTION_RANGE=   30 0.8

4. Direct beam position
 ORGX= 258  ORGY=  258

5. Camera length
 DETECTOR_DISTANCE=  +439.48

6. Oscillation range, degree per frame:
 OSCILLATION_RANGE= 0.23

7. Rotation axis, depending on microscope:
 ROTATION_AXIS= -0.6203 0.7843 0  !cos(rotation_axis) cos(axis-90)  !in XDS.INP

8. Wavelength, Å (200 keV 0.02508, 300 keV 0.01968):
 X-RAY_WAVELENGTH=  0.0251     !used by IDXREF

###Additional Keywords###
UNTRUSTED_RECTANGLE= 255 262 0 517
UNTRUSTED_RECTANGLE= 0 517 255 262
###Additional Keywords###
```

S3.3 Realtime Strategy File:

```
! CYCLE_TIME, refresh time, default 5 s.
! WAITING_TIME, time waiting for real processing
! after folder is unchanged, default 10 s.
 CYCLE_TIME= 5
 WAITING_TIME= 20

! Available Filter for RUN_FILTER: CC12, ISA, REXP, RMEAS, RESOLUTION, VOLUME_DEV,
unit in %
 RUN_FILTER= CC12 > 85
 RUN_FILTER= ISA > 2
 RUN_FILTER= ISA < 50

! Available Filter for MERGE_FILTER: CC12, DISTANCE
 MERGE_FILTER= CC12 > 0
```

S4. AutoLEI graphic user interface

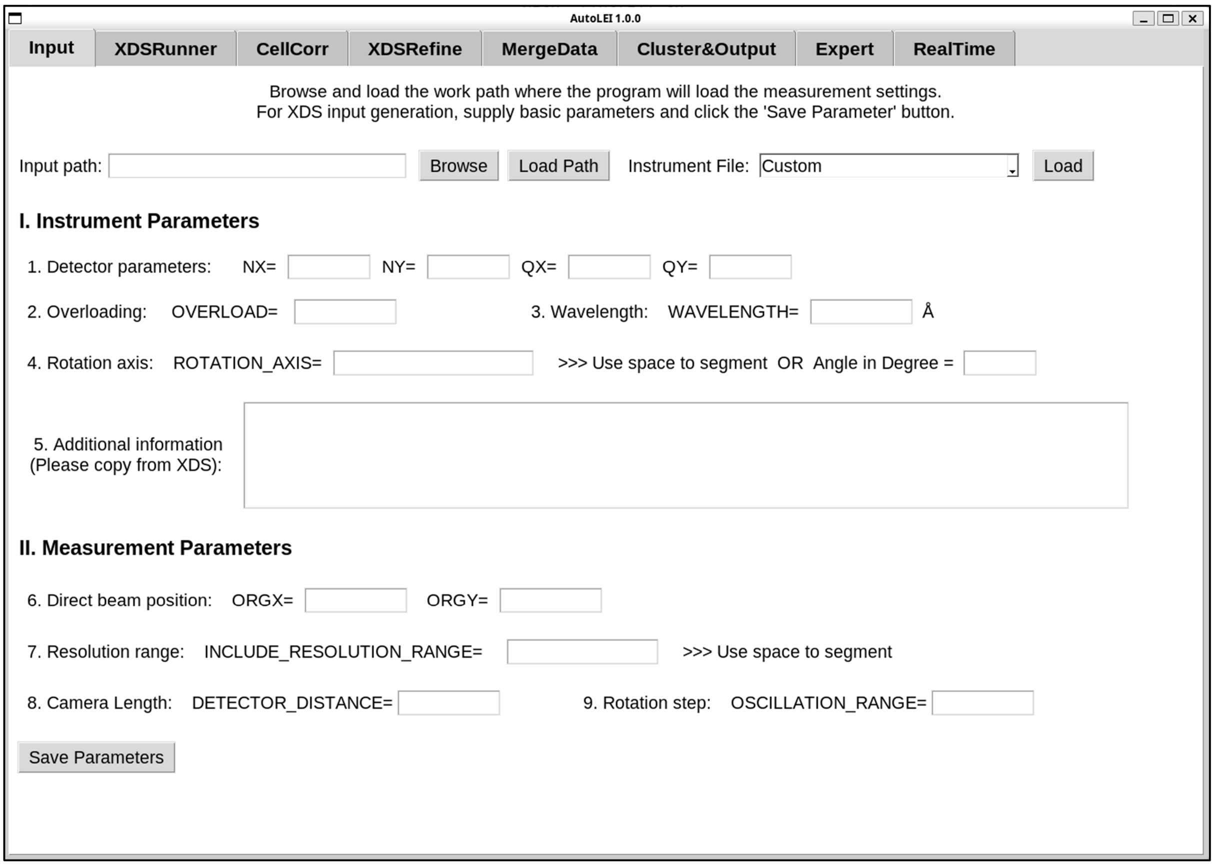

Figure S1 AutoLEI interface of ***Input***

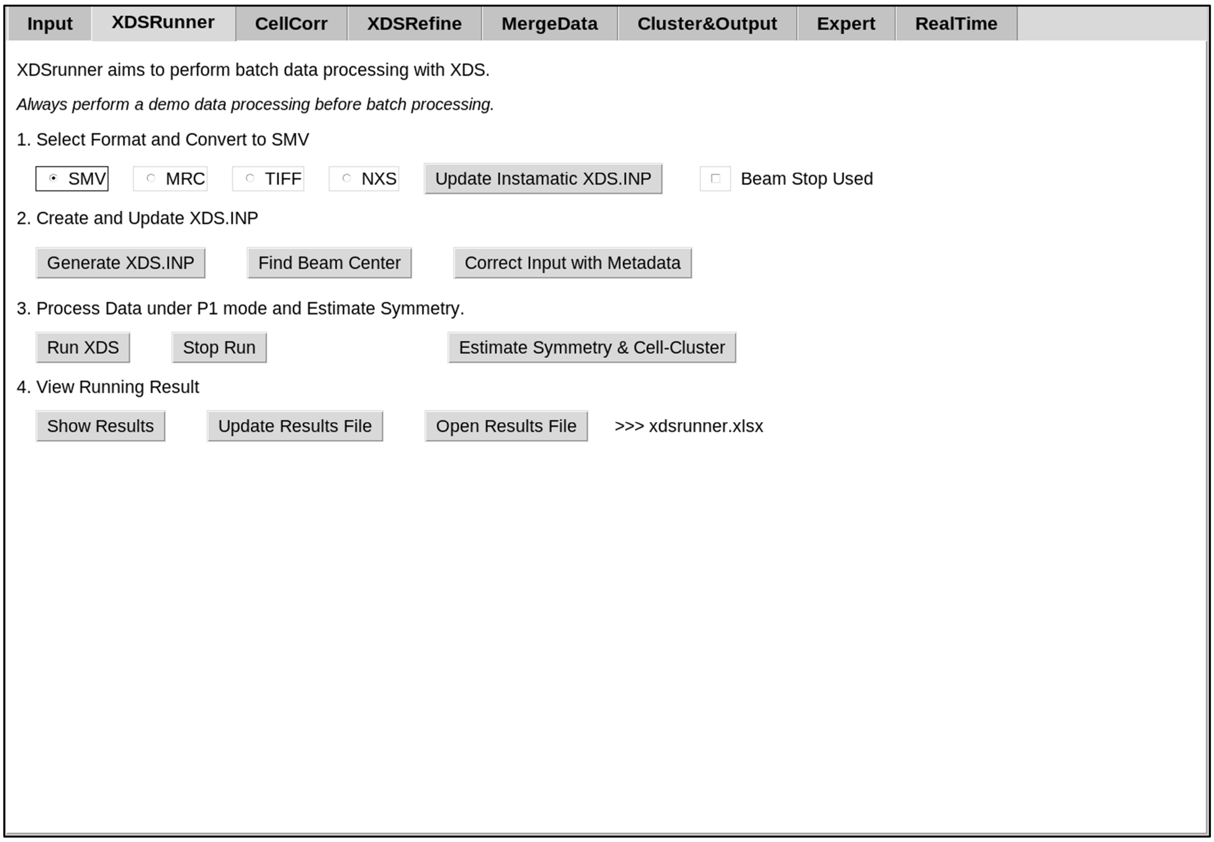

Figure S2 AutoLEI interface of ***XDSRunner***

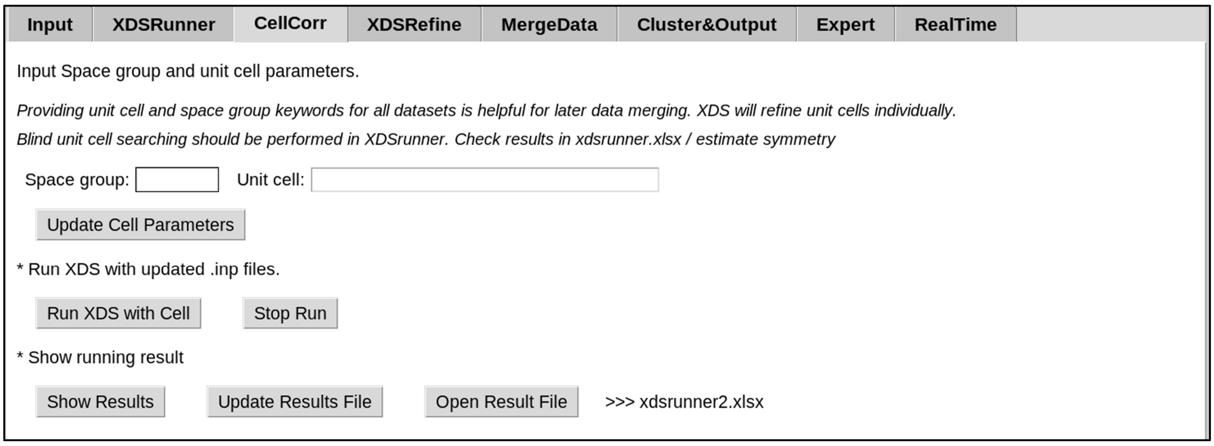

Figure S3 AutoLEI interface of ***CellCorr***

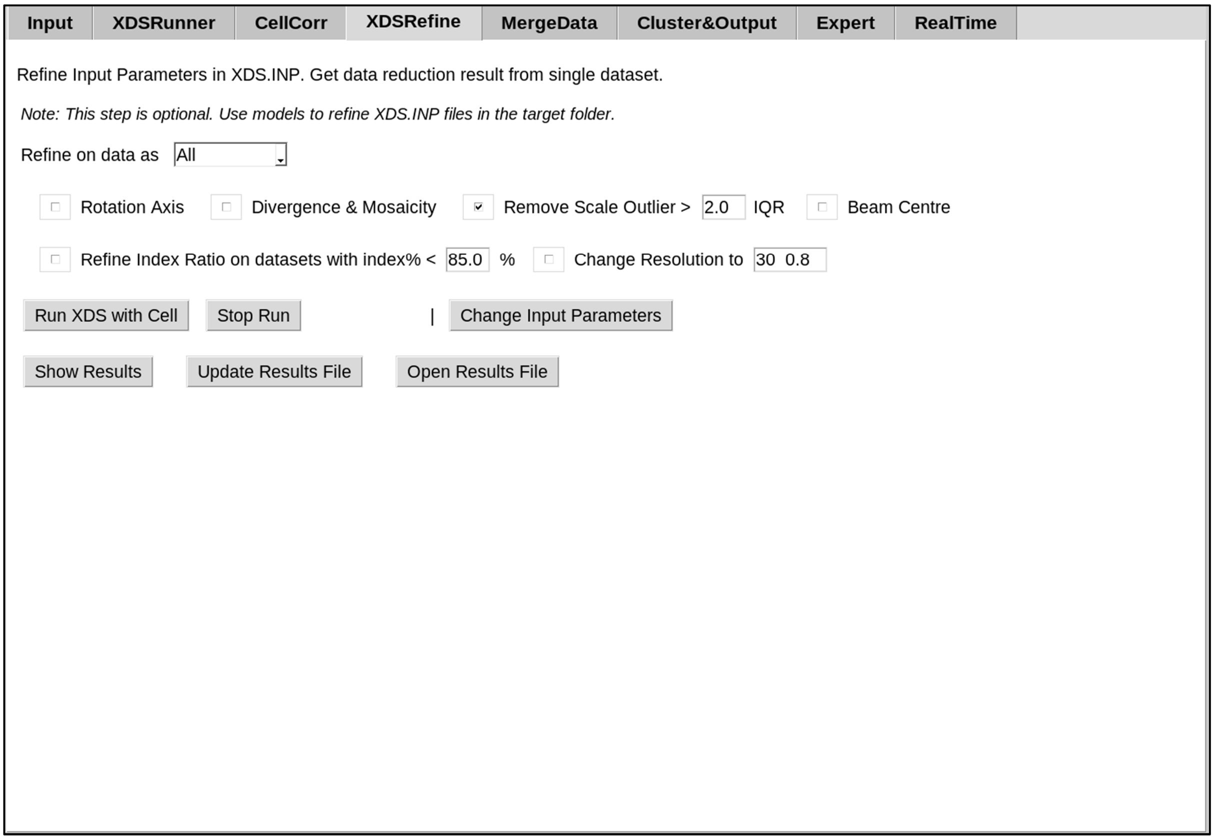

Figure S4 AutoLEI interface of ***XDSRefine***

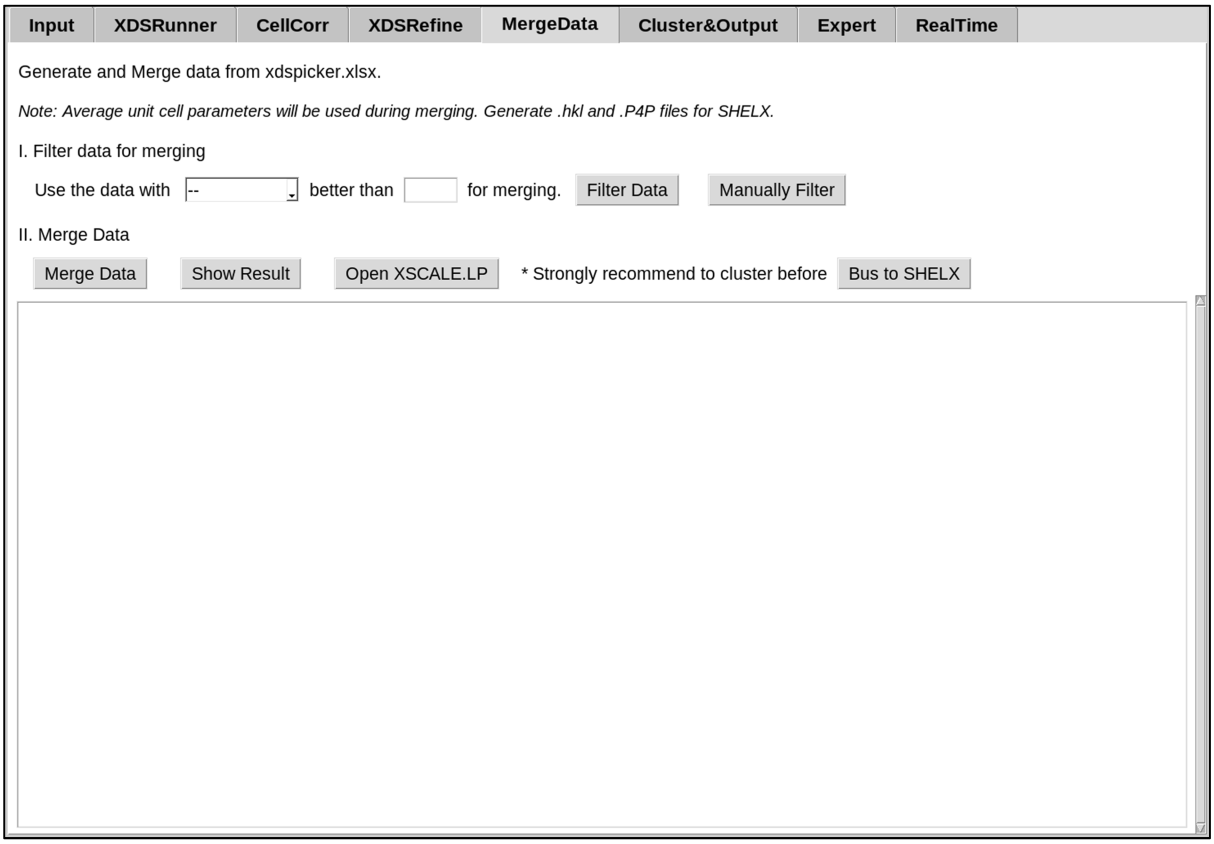

Figure S5 AutoLEI interface of ***MergeData***

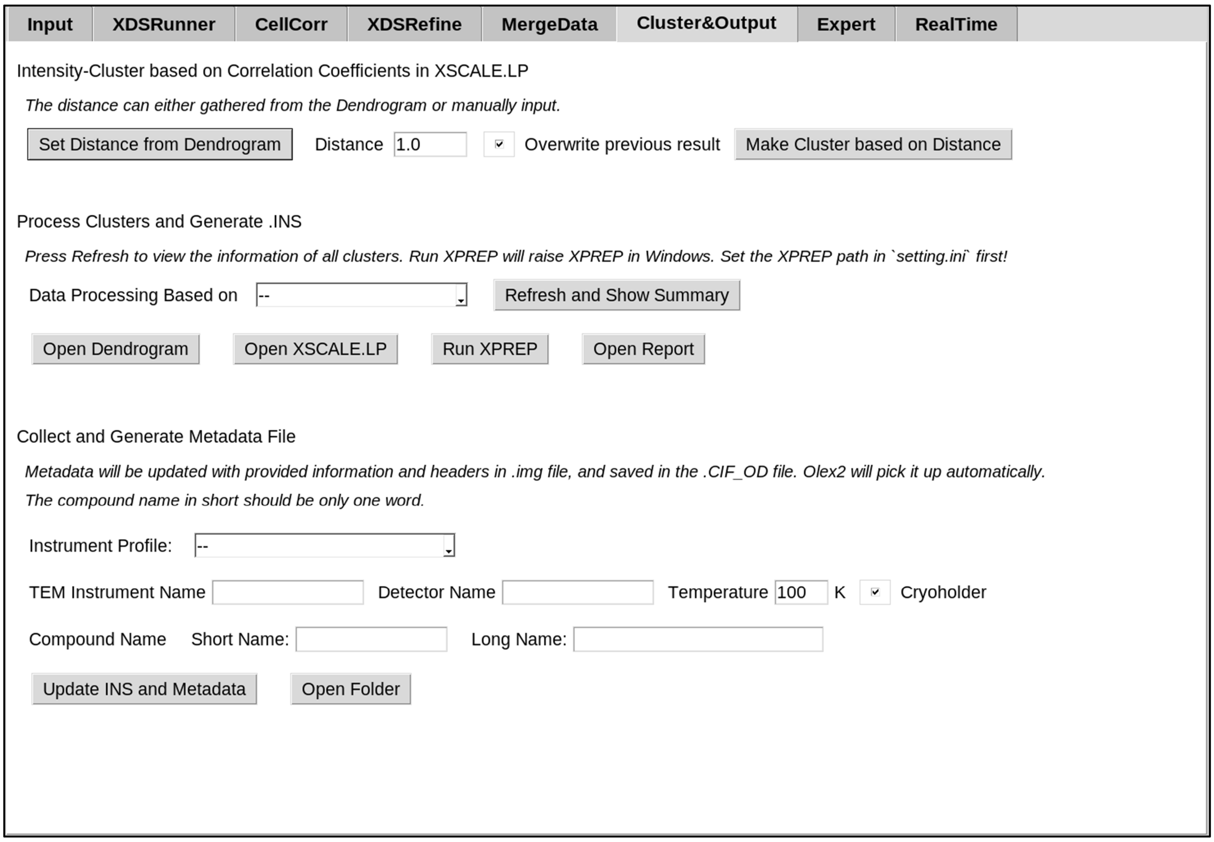

Figure S6 AutoLEI interface of ***Cluster***

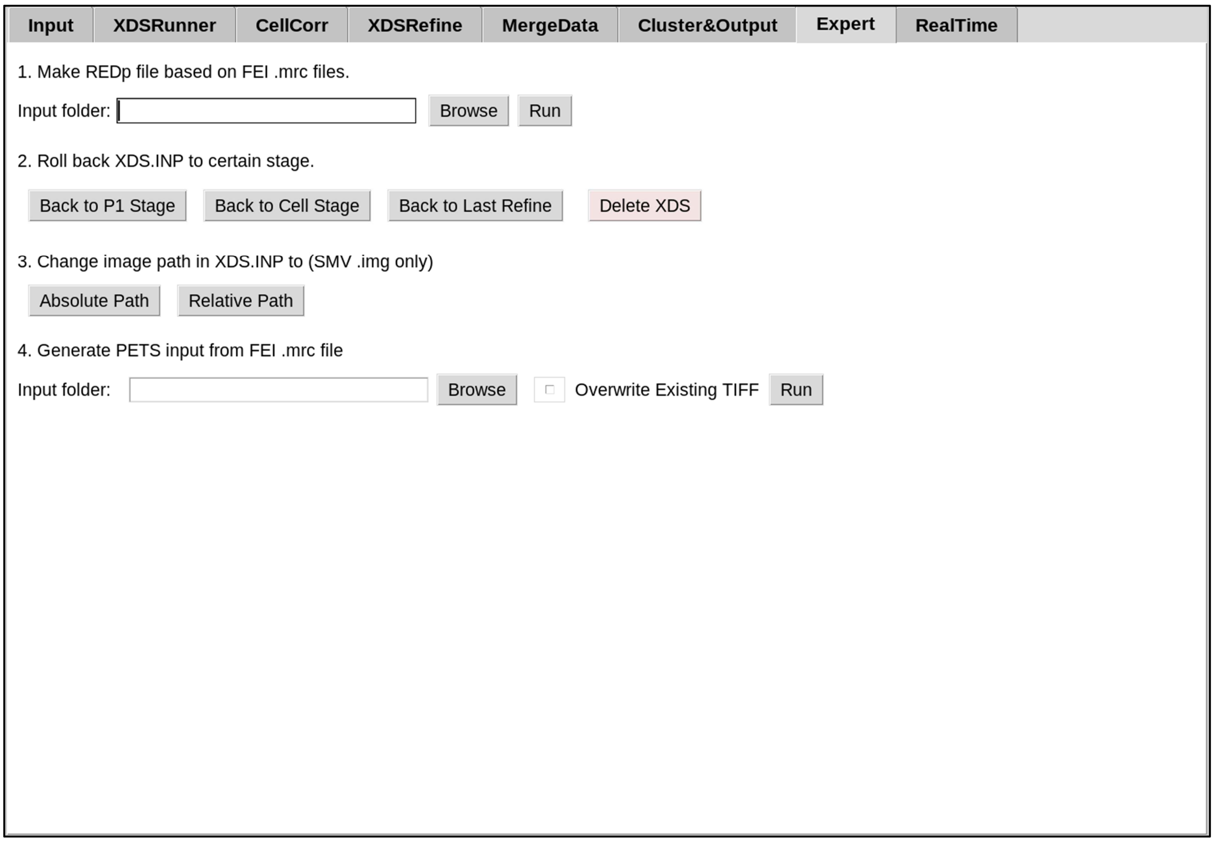

Figure S7 AutoLEI interface of ***Expert***

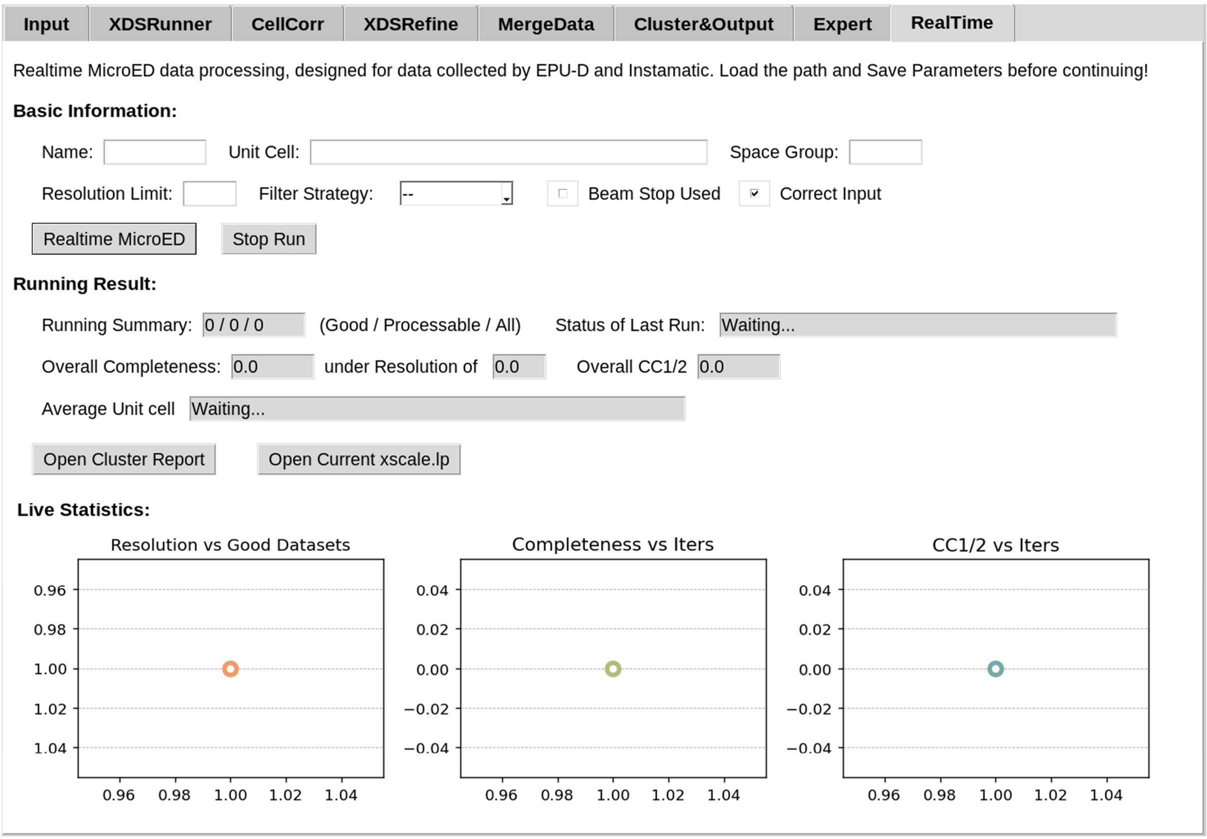

Figure S8 AutoLEI interface of ***RealTime***

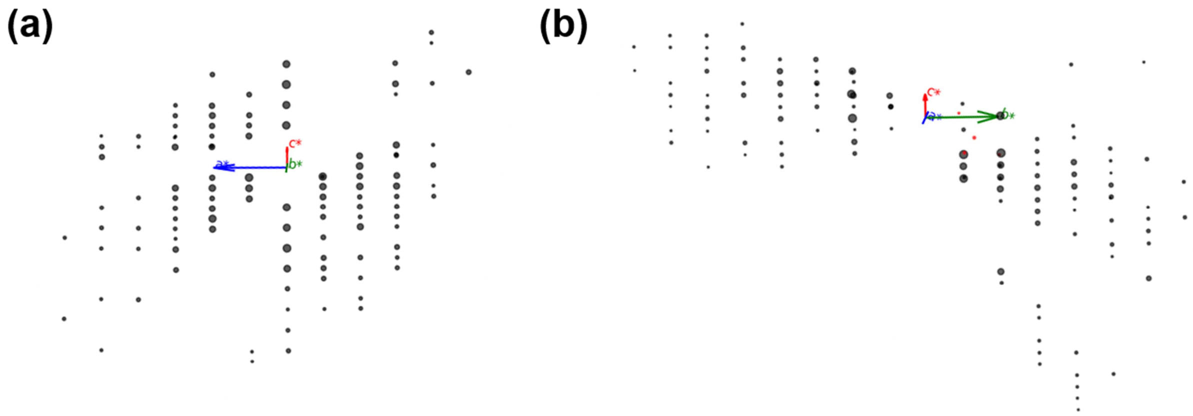

Figure S9 2D slices of 3D reciprocal lattice of L-tyrosine showing (a) $h0l$ plane reconstructed from 3D ED/MicroED data of Experiment_10 and (b) $0kl$ plane reconstructed from 3D ED/MicroED data from Experiment_12. The reflection conditions could be deduced as $h0l$ and $0kl$: no conditions, $h00$: $h = 2n$, $0k0$: $k = 2n$ and $00l$: $l = 2n$, corresponding to the space group $P2_12_12_1$.

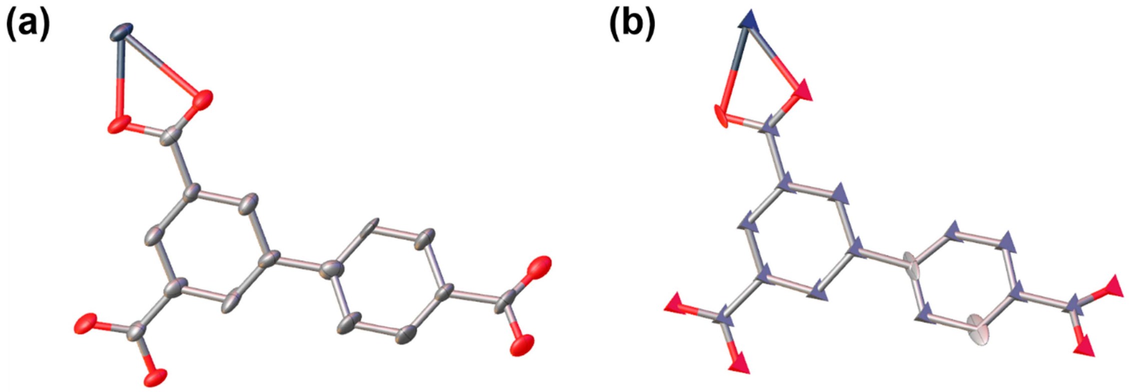

Figure S10 SU-100 structure refined against (a) data merged from 11 datasets and (b) a single dataset (experiment_2). While the atomic displacement parameters are reasonable in (a), most atoms have non-positively defined atomic displacement parameters (indicated by tetrahedra in (b)). Hydrogen atoms are omitted for clarity. Atom colors are: bismuth (greyish blue), carbon (grey), and oxygen (red).

S5. Crystallographic tables of the four different samples

Table S1 Crystallographic data and refined details of 12 tyrosine datasets

No.	Resolution (Å)	Completeness (%)	ISa	R_{meas} (%)	$CC_{1/2}$ (%)	Space group	*a* (Å)	*b* (Å)	*c* (Å)	No. of reflections (obs/unique/all)	R_{int}	No. of parameters	No. of restraints	R_1 (obs)	R_1 (all)	wR$_2$ (all)	GOOF
1	0.56	60.01	9.33	15.38	99.10	$P2_12_12_1$	5.994(8)	7.076(4)	21.745(4)	1560/2933/5277	0.1140	162	8	0.1578	0.2059	0.4246	1.220
2	0.55	57.16	11.5	12.79	99.10	$P2_12_12_1$	5.997(6)	7.035(5)	21.796(9)	1706/2867/5390	0.0940	151	14	0.1587	0.2076	0.4041	1.270
3	0.62	33.46	7.25	14.58	97.33					No solution							
4	0.56	50.29	7.45	17.07	98.70	$P2_12_12_1$	5.997(12)	6.984(3)	21.761(7)	1272/2453/4993	0.1331	162	10	0.1398	0.1955	0.3756	1.048
5*	0.74	74.37	5.09	24.64	96.37	$P2_12_12_1$	6.011(16)	7.029(6)	21.75(6)	1178/2477/5132	0.1900	54	0	0.2036	0.2766	0.4854	1.198
6	0.56	49.63	6.33	22.16	97.30	$P2_12_12_1$	5.998(19)	6.981(14)	21.732(17)	1063/2324/3581	0.1843	158	21	0.1504	0.2125	0.4027	1.003
7	0.93	5.64								Data Reduction Failed							
8	0.54	58.21	6.42	18.45	97.80	$P2_12_12_1$	6.017(16)	7.049(5)	21.752(14)	1761/3204/5475	0.1399	158	34	0.1605	0.2059	0.4001	1.093
9*	0.54	53.01	8.31	14.61	99.10	$P2_12_12_1$	5.995(9)	6.957(13)	21.827(16)	1343/2694/4938	0.1089	55	0	0.2161	0.3065	0.5664	1.666
10	0.68	87.61	7.51	19.45	98.44	$P2_12_12_1$	6.0080(12)	7.0250(14)	21.690(4)	1253/2592/6260	0.1558	162	8	0.1190	0.1794	0.3192	0.995
11	0.56	45.46	11.15	11.18	98.8	$P2_12_12_1$	5.988(4)	7.193(13)	21.733(5)	1481/2100/3921	0.0881	143	10	0.1596	0.1857	0.4111	1.419
12	0.56	85.16	7.19	24.21	97.60	$P2_12_12_1$	6.010(17)	7.050(7)	21.77(5)	1750/3199/6277	0.1892	162	13	0.1505	0.1955	0.3917	1.083

* Isotropic refinement for non-hydrogen atoms.

Table S2 Crystallographic data and refined details of SU-100

**SU-100**	
Chemical Formula	$C_{15}H_7BiO_6$
No. datasets	11
Wavelength (Å)	0.02508
Temperature (K)	100
Space group	*C*2/*c*
a (Å)	26.44(3)
b (Å)	10.049(17)
c (Å)	18.135(17)
β (°)	127.28(9)
Volume (Å^3)	3834(9)
Z	8
Completeness (%, to 0.84/0.62 Å)	94.9/85.4
Resolution (Å)	0.62
R_{meas} (%)	44.3
$CC_{1/2}$ (%)	97.8
R_{int}	0.3566
No. unique reflections (obs/all)	4231/7193
No. total reflections	101819
No. parameters	200
No. restraints	0
R_1 (obs)	0.1946
R_1 (all)	0.2572
wR_2 (all)	0.4865
GOOF	1.103

Table S3 Data collection and refinement statistics of MTH1 and lysozyme

**Sample**	**apo-MTH1**	**Lysozyme**
**Data processing**		
No. of crystals	18	56
Space group	$P2_12_12_1$	$P1$
a, b, c (Å)	61.66, 69.71, 80.87	26.39, 30.98, 33.26
α, β, γ (°)	90, 90, 90	88.298, 108.613, 111.985
Resolution (Å)	23.13 – 2.76 (2.91 – 2.76)	10.53 – 1.1 (1.12 – 1.10)
No. of observations	105667 (6554)	238462 (6691)
No. of unique reflections	8449 (1010)	34045 (1454)
$CC_{1/2}$ (%)	97.9 (42.8)	98.6 (31.5)
R_{merge} (%)	44.2 (151.8)	30.8 (118.9)
R_{meas} (%)	45.9 (164.0)	33.1 (134.1)
R_{pim} (%)	11.6 (56.2)	11.3 (58.3)
Multiplicity	12.5 (6.5)	7.0 (4.6)
Completeness (%)	90.9 (75.2)	91.1 (78.7)
**Refinement**		
No. unique reflections	8397	34030
R_{work}/R_{free} (%)	21.07/26.82	20.42/23.45
Mean B-factors ($Å^2$)	39.01	9.68
R.M.S. deviations		
Bond lengths (Å)	0.002	0.023
Bond angle (°)	0.549	1.484
Ramachandran		
Favored (%)	95.08	98.40
Allowed (%)	4.59	1.6
Outliers (%)	0.33	0
Clashscore	2.01	7.21
Rotamer outliers (%)	1.11	0

Values in parentheses are for the highest-resolution shell.

Reference

Afonine, P. V., Grosse-Kunstleve, R. W., Echols, N., Headd, J. J., Moriarty, N. W., Mustyakimov, M., Terwilliger, T. C., Urzhumtsev, A., Zwart, P. H. & Adams, P. D. (2012). *Acta Crystallogr D Biol Crystallogr* **68**, 352–367.

Burnley, T., Palmer, C. M. & Winn, M. (2017). *Acta Crystallogr D Struct Biol* **73**, 469–477.

Cichocka, M. O., Ångström, J., Wang, B., Zou, X. & Smeets, S. (2018). *J Appl Crystallogr* **51**, 1652–1661.

Dolomanov, O. V., Bourhis, L. J., Gildea, R. J., Howard, J. A. K. & Puschmann, H. (2009). *J Appl Crystallogr* **42**, 339–341.

Giordano, R., Leal, R. M. F., Bourenkov, G. P., McSweeney, S. & Popov, A. N. (2012). *Acta Crystallogr D Biol Crystallogr* **68**, 649–658.

Grant, J. A. & Pickup, B. T. (1995). *J. Phys. Chem.* **99**, 3503–3510.

Grape, E. S., Xu, H., Cheung, O., Calmels, M., Zhao, J., Dejoie, C., Proserpio, D. M., Zou, X. & Inge, A. K. (2020). *Crystal Growth & Design* **20**, 320–329.

Grosse-Kunstleve, R. W. (1999). *Acta Crystallogr A Found Crystallogr* **55**, 383–395.

Grosse-Kunstleve, R. W., Sauter, N. K. & Adams, P. D. (2004). *Acta Crystallogr A Found Crystallogr* **60**, 1–6.

Jain, A. K. & Dubes, R. C. (1988). Algorithms for Clustering Data Prentice Hall.

Knudsen, E. B., Sørensen, H. O., Wright, J. P., Goret, G. & Kieffer, J. (2013). *J Appl Crystallogr* **46**, 537–539.

Kolb, U., Shankland, K., Meshi, L., Avilov, A. & David, W. I. F. (2012). Uniting Electron Crystallography and Powder Diffraction Dordrecht: Springer Netherlands.

Liebschner, D., Afonine, P. V., Baker, M. L., Bunkóczi, G., Chen, V. B., Croll, T. I., Hintze, B., Hung, L.-W., Jain, S., McCoy, A. J., Moriarty, N. W., Oeffner, R. D., Poon, B. K., Prisant, M. G., Read, R. J., Richardson, J. S., Richardson, D. C., Sammito, M. D., Sobolev, O. V., Stockwell, D. H., Terwilliger, T. C., Urzhumtsev, A. G., Videau, L. L., Williams, C. J. & Adams, P. D. (2019). *Acta Crystallogr D Struct Biol* **75**, 861–877.

McCoy, A. J., Grosse-Kunstleve, R. W., Adams, P. D., Winn, M. D., Storoni, L. C. & Read, R. J. (2007). *J Appl Crystallogr* **40**, 658–674.

Niggli, P. (1928). Vol. *7*, *Handbuch Der Experimentalphysik*. pp. 108–176. Leipzig: Akademische Verlagsgesellschaft.

Palatinus, L. (2013). *Acta Crystallogr B Struct Sci Cryst Eng Mater* **69**, 1–16.

Schönemann, P. H. (1966). *Psychometrika* **31**, 1–10.

Sheldrick, G. M. (2008). *Acta Crystallogr A Found Crystallogr* **64**, 112–122.

Storn, R. & Price, K. (1997). *Journal of Global Optimization* **11**, 341–359.

Svensson, L. M., Jemth, A.-S., Desroses, M., Loseva, O., Helleday, T., Högbom, M. & Stenmark, P. (2011). *FEBS Letters* **585**, 2617–2621.

Van Der Walt, S., Schönberger, J. L., Nunez-Iglesias, J., Boulogne, F., Warner, J. D., Yager, N., Gouillart, E. & Yu, T. (2014). *PeerJ* **2**, e453.

Wang, J., Dauter, M., Alkire, R., Joachimiak, A. & Dauter, Z. (2007). *Acta Crystallogr D Biol Crystallogr* **63**, 1254–1268.
